# A coronaviral pore-replicase complex links RNA synthesis and export from double-membrane vesicles

**DOI:** 10.1126/sciadv.adq9580

**Published:** 2024-11-08

**Authors:** Anan Chen, Ana-Mihaela Lupan, Rui Tong Quek, Stefan G. Stanciu, Mihaela Asaftei, George A. Stanciu, Kierra S. Hardy, Taciani de Almeida Magalhães, Pamela A. Silver, Timothy J. Mitchison, Adrian Salic

**Affiliations:** ^1^ Department of Cell Biology, Harvard Medical School, Boston, MA 02115, USA.; ^2^ Department of Systems Biology, Harvard Medical School, Boston, MA 02115, USA.; ^3^ Center for Microscopy-Microanalysis and Information Processing, National University of Science and Technology Politehnica Bucharest, 313 Splaiul Independenței, 060042 Bucharest, Romania.; ^4^ Department of Microbiology, University of Bucharest, Aleea Portocalelor nr. 1-3, 060101 Bucharest, Romania.; ^5^ Wyss Institute for Biologically Inspired Engineering, Harvard University, Boston, MA 02115, USA.; ^6^ Faculty of Chemistry, University of Bucharest, Șoseaua Panduri nr. 90, 050663 Bucharest, Romania.

## Abstract

Coronavirus-infected cells contain double-membrane vesicles (DMVs) that are key for viral RNA replication and transcription, perforated by hexameric pores connecting the vesicular lumen to the cytoplasm. How pores form and traverse two membranes, and how DMVs organize RNA synthesis, is unknown. Using structure prediction and functional assays, we show that the nonstructural viral membrane protein nsp4 is the key pore organizer, spanning the double membrane and forming most of the pore lining. Nsp4 interacts with nsp3 on the cytoplasmic side and with the viral replicase inside the DMV. Newly synthesized mRNAs exit the DMV into the cytoplasm, passing through a narrow ring of conserved nsp4 residues. Steric constraints imposed by the ring predict that modified nucleobases block mRNA transit, resulting in broad-spectrum anticoronaviral activity.

## Teaser

Coronaviral nsp4 is the key organizer of double-membrane vesicle pores and forms a complex with the active viral replicase.

## INTRODUCTION

Coronaviruses have large single-stranded (ss) RNA genomes (up to 33 kb) (*1*) that encode a polyprotein followed by four structural and several accessory proteins. The polyprotein is cleaved into 16 nonstructural proteins (nsps) (*2*) that play key roles in the viral takeover of the infected cell. Nsps 7, 8, 9, 12, and 13 form the RNA-dependent RNA polymerase complex (RdRp or replicase), which first copies and then transcribes the viral genome, of which nsp12 is the catalytic subunit (*3*–*5*). The three transmembrane nsps 3, 4, and 6 remodel endoplasmic reticulum (ER) membranes into characteristic double-membrane vesicles (DMVs). DMVs are thought to play a central role in viral RNA synthesis and to protect the double-stranded RNA (dsRNA) replication intermediate from recognition by cytoplasmic innate immune sensors (*6*–*9*). DMVs have an inner lumen containing filament-like structures (*10*), identified as dsRNAs that represent viral genome replication intermediates (*10*, *11*). The lumen is connected to the host cytoplasm via sixfold symmetric pores that contain nsp3 (*12*, *13*) and are thought to mediate the exchange of molecules between the two compartments. Other viral nsps, such as nsp4 and nsp8, have been localized in the proximity of DMVs or in adjacent membranous structures, by light microscopy or immuno–electron microscopy (*11*, *14*, *15*). However, the molecular nature of the DMV pores, the relationship between the replicase and DMVs, and the precise role of DMVs in viral RNA synthesis remain unknown. Pioneering cryo–electron microscopy (cryo-EM) tomography studies in mouse hepatitis virus–infected cells showed that six copies of nsp3 form the cytoplasm-facing aspect of the DMV pore (*12*). However, nsp3 contains only two transmembrane helices (TMs), suggesting that a nsp3 hexamer alone would be insufficient on its own to delineate a pore that is more than 2 nm in diameter at its narrowest and traverses two phospholipid bilayers that are 10 to 11 nm apart (*12*).

## RESULTS

**Fig. 1.**
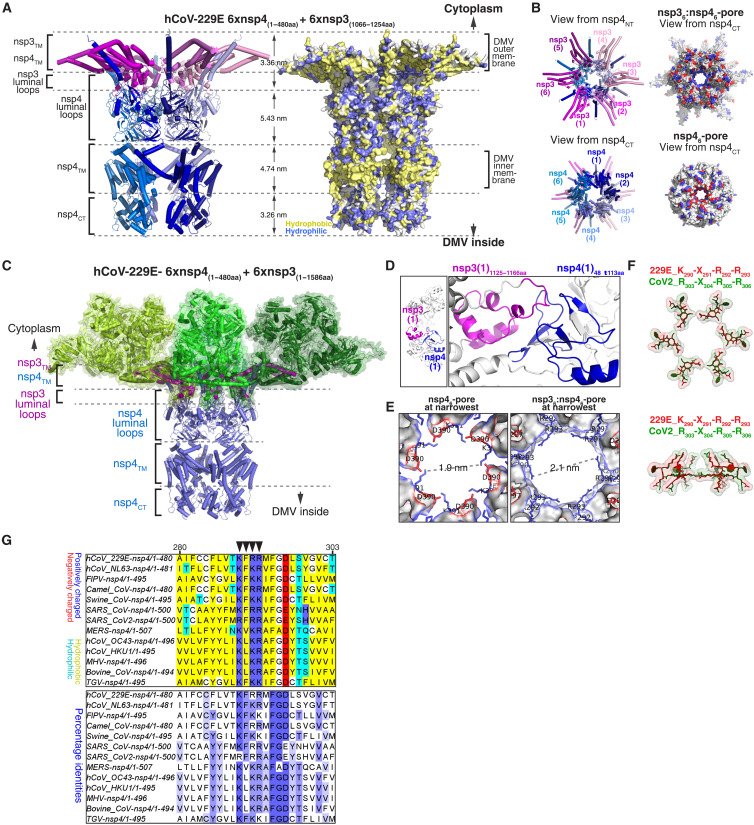
A coronaviral nsp3-nsp4 complex defines the DMV molecular pore. (**A**) Predicted structure of a sixfold symmetric complex between hCoV-229E nsp3 (residues 1066 to 1254) and full-length nsp4, seen from the side. TM, transmembrane domains; NT, N terminus; CT, C terminus. Left: Ribbon diagram. Right: Molecular surface showing hydrophobic (yellow) and hydrophilic (blue) residues. Distances between domains are indicated. (**B**) As in (A), but showing top and bottom views of the complex, respectively. Left: Ribbon diagram. Right: Molecular surface showing positive (blue) and negative (red) residues. (**C**) As in (A), but including the predicted structure of full-length nsp3 (green). (**D**) Close-up view of the interaction between the intermembrane domains of hCoV-229E nsp3 and nsp4. (**E**) Predicted narrowest portion of the pore formed by hCoV-229E nsp4 alone (left) or in complex with nsp3 (right). The diameter of the constriction and the conserved residues delineating it are indicated. (**F**) As in (E), but showing the overlay of the conserved pore constriction residues in hCoV-229E (red) and severe acute respiratory syndrome coronavirus 2 (green). (**G**) Sequence alignment of nsp4 homologs from various α- or β-coronaviruses, showing the conserved K-X-R/K-R/K pore motif (arrows).

We reasoned that nsp4, which has six TMs, is a good candidate for the main coronaviral pore-forming protein. ColabFold multimer (*16*, *17*) predicted with high confidence that full-length nsp4 assembles into a symmetric hexameric complex with a continuous central tunnel (fig. S1A). This predicted structure is highly conserved among coronavirus species, despite modest sequence conservation among nsp4 orthologs (fig. S1B). The tunnel’s smallest diameter is 1.9 nm (Fig. 1E), slightly less than the diameter measured for DMV pores in cells (*12*). Because nsp4 interacts with nsp3 (*18*–*20*), we next obtained a high-confidence model of a complex containing six copies each of full-length nsp4 and an N-terminally truncated nsp3 (ΔN-nsp3), a fragment including the two TMs, and the intervening luminal domain that interacts with nsp4 (*18*–*20*); ΔN-nsp3 and full-length nsp4 suffice for DMV formation in cells (Fig. 1, A and B). We then aligned six copies of the predicted structure of full-length nsp3 (Fig. 1C and fig. S1, C to E), which could be fitted into the model without steric clashes. The resulting sixfold symmetric nsp3-nsp4 complex (Fig. 1C) is notably similar in size and shape to the DMV pore determined by cryo-EM tomography (*12*). The intermembrane distance for the predicted nsp3-nsp4 complex (16.8 nm) is comparable to previous cryo-EM tomography measurements of the pore (16 to 18 nm) (*10*, *13*). Like the nsp4 hexamer, the nsp3-nsp4 complex also has a central tunnel, the narrowest portion being now increased to 2.1 to 2.3 nm (Fig. 1, B, E, and F, and fig. S1F), in good accord with DMV pore measurements in virus-infected cells (*12*). This constriction is delineated by a ring of 18 positively charged residues contributed by the six nsp4 molecules (Fig. 1, E and F, and fig. S1F); these residues form a consensus (-K/R_290_-X_291_-R/X_292_-R/K_293_-) that is highly conserved among coronaviral nsp4 orthologs (Fig. 1, F and G).

A major unanswered question is how the pore spans the two closely spaced DMV membranes. In our model, the last five of the six TMs of nsp4 localize to the inner membrane (Fig. 1, A and C), while the ER-luminal domain forms most of the portion of the pore spanning the intermembrane space (Fig. 1A). The model predicts an interface between the ER-luminal domains of nsp3 and nsp4 (Fig. 1D), in accord with longstanding biochemical data (*18*–*20*). The N-terminal TM of nsp4 lies on the opposite side of the molecule from the other TMs (Fig. 1A and fig. S1A), as previously hypothesized (*13*), and localizes to the outer DMV membrane together with the two TMs of nsp3. Last, the soluble N- and C-terminal domains (NTD and CTD) of nsp3 form the entire cytoplasm-facing portion of the pore (Fig. 1C and fig. S1D). Thus, our model suggests that the DMV pore consists of a hexameric nsp3-nsp4 complex and that nsp4 plays a key role in spanning the double membrane.

**Fig. 2.**
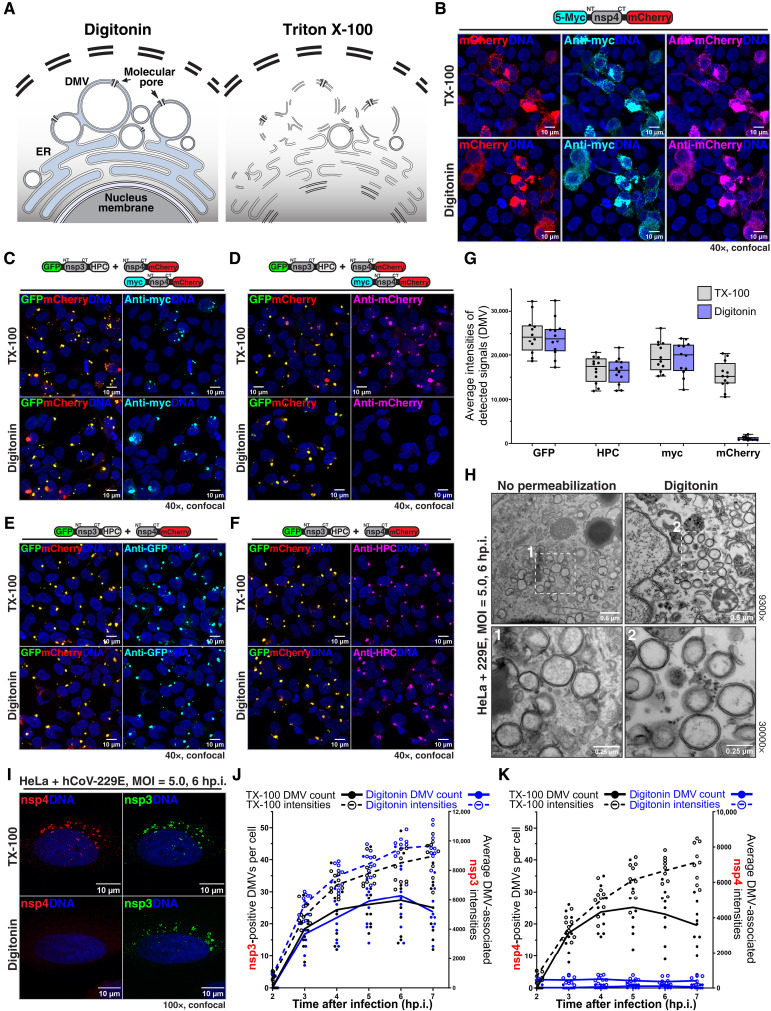
Nsp4 spans both DMV membranes, extending from the cytoplasm to the lumen. (**A**) Differential antibody accessibility assay. Fixed cells permeabilized with digitonin or Triton X-100 are subjected to immunofluorescence staining. (**B**) In cells expressing myc-nsp4-mCherry, both myc and mCherry tags face the cytoplasm. The experiment was repeated three times on 3 days. (**C** and **D**) As in (B), but with cells coexpressing eGFP-nsp3ΔN-HPC and myc-nsp4-mCherry. The myc tag now faces the cytoplasm, while the mCherry tag faces the DMV lumen. (**E** and **F**) As in (C), but stained with indicated antibodies. (**G**) Quantification of experiments in (C) to (F). Four nonoverlapping fields were analyzed per condition and each condition was repeated thrice. (*N* = 3, *n* = 12). (**H**) Transmitted electron microscopy imaging of hCoV-229E–infected cells [multiplicity of infection (MOI) = 5.0] showing viral DMVs at 6 hp.i. Close-ups of the outlined regions are shown at the bottom. (**I**) As in (H), but cells were differentially permeabilized and costained with antibodies against nsp3-NT and nsp4-CT. Nsp3-NT faces the cytoplasm while nsp4-CT faces the DMV lumen. (**J** and **K**) As in (I), showing quantification of nsp3 (J) or nsp4 (I) differential accessibility at different time points after infection. Three nonoverlapping fields of view were analyzed per condition and each condition was repeated thrice. (*N* = 3, *n* = 9).

A key prediction of our model is that the N and C termini of nsp3 face the cytoplasm, while those of nsp4 face the cytoplasm and the DMV lumen, respectively (Fig. 1C and fig. S1D). To test experimentally this topology, we first used a reconstituted cellular system in which coexpression of ΔN-nsp3 and nsp4 generates abundant synthetic DMVs ~100 nm in diameter (fig. S2, A and B), in the absence of viral infection. Under these conditions, ΔN-nsp3 and nsp4 show strong colocalization and interaction (fig. S2, A and D). The synthetic DMVs and those formed during hCoV-229E infection (Fig. 2H) have an identical intermembrane distance (15.7 ± 2.8 nm versus 16.1 ± 3.3 nm; fig. S2C); this distance fits well with the intermembrane distance of the predicted nsp3-nsp4 complex (16.8 nm; Fig. 1A). We then used differential permeabilization and immunofluorescence to probe antibody accessibility to tags attached to the N and C termini of nsp3 and nsp4. In the absence of nsp3, both N and C termini of nsp4 were equally accessible (Fig. 2B), whether the cells were treated with digitonin, which permeabilizes the plasma membrane but not intracellular membranes, or with Triton X-100 (TX-100), which permeabilizes all membranes (Fig. 2, B and H, and fig. S3A). As expected, a nuclear-localized protein was accessible to antibodies only after TX-100, but not digitonin, permeabilization (fig. S3, B and C). Thus, both nsp4 termini face the cytoplasm when the protein is expressed alone. However, when synthetic DMVs were induced by coexpressing nsp3, the nsp4 C terminus became inaccessible with digitonin, while accessibility of the N terminus was unchanged (Fig. 2, C, D, and G, and fig. S3, F and G). The N and C termini of nps3 were both accessible with digitonin permeabilization (Fig. 2, C, D, and G, and fig. S3, H and I). Similar results were obtained using synthetic DMVs immunopurified via a C-terminal tag on nsp3 (figs. S2, E to H, and S3, D and E). Consistent with antibody accessibility results, we found that an N-terminal tag on nsp3 is rapidly degraded by proteinase K under digitonin permeabilization conditions, while a C-terminal tag on nsp4 is protected (fig. S4). Last, we tested antibody accessibility in DMVs formed during hCoV-229E infection, using affinity-purified antibodies against the NTD of nsp3 and the CTD of nsp4, and obtained identical results to those in synthetic DMVs (Fig. 2, H to K). Together, these data confirm the predicted topology of the nsp3-nsp4 complex: Both nsp3 termini face the cytoplasm, while the N and C termini of nsp4 face the cytoplasm and DMV lumen, respectively. Furthermore, the results suggest that nsp4 plays a central role in pore assembly and DMV closure.

**Fig. 3.**
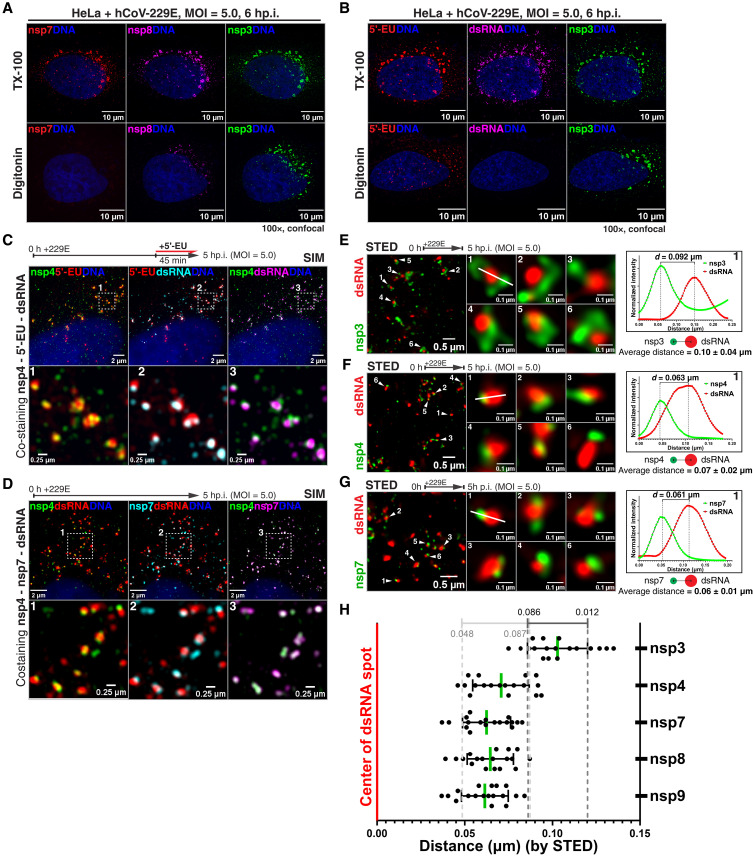
The viral replicase localizes inside DMVs, in close proximity to nsp4. (**A** and **B**) hCoV-229E–infected cells (MOI = 5.0, 6 hp.i.) labeled with EU were fixed and permeabilized, then (A) stained with fluorescently labeled antibodies against nsp7, nsp8, and nsp3 or (B) reacted by click chemistry with biotin-azide, followed by costaining with fluorescent streptavidin, dsRNA probe, and anti-nsp3 antibodies. Nsp7 and dsRNA are inside DMVs, while EU-labeled transcripts are both inside DMVs and cytoplasmic. The experiment was repeated three times in three different days. (**C**) As in (A) and (B), but cells fixed at 5 hp.i. with staining for nsp4 and imaging by SIM. (**D**) As in (C), but with costaining for nsp4, nsp7 and dsRNA. (**E** to **G**) hCoV-229E–infected cells (MOI = 5.0, 5 hp.i.) were fixed, permeabilized, and costained for (E) nsp3 and dsRNA, (F) nsp4 and dsRNA, or (G) nsp7 and dsRNA, followed by imaging by STED microscopy. Middle panels show zoomed-in views of DMVs on the left. The white line in middle panel 1 was used for linescan analysis (right), to measure distance between nsps and dsRNA. (**H**) Quantification of center-to-center distance between nsps and dsRNA in (G). Eighteen zoomed-in images from three nonoverlapping fields were analyzed for each group (*n* = 18). Nsp4, nsp7, nsp8, and nsp9 localize closer to dsRNA, while nsp3 is further away.

We extended the accessibility analysis to localize dsRNA in cells infected with hCoV-229E, using affinity-purified antibodies against viral proteins and a probe we developed based on protein kinase R (PKR) that detects dsRNA with high sensitivity (Fig. 3 and fig. S5). dsRNA and viral proteins were detected as early as 2 hours after infection (Fig. 3, A and B, and fig. S5, B to F). At all times, dsRNA and the core RdRp subunit nsp7 were inaccessible under digitonin permeabilization conditions (Fig. 3, A and B, and fig. S5, B and D), similar to the C terminus of nsp4, while two other RdRp subunits, nsp8 and nsp9, were only weakly accessible (Fig. 3A and fig. S5, A, E, and F); all these components were accessible with TX-100 permeabilization, showing that the viral dsRNA and the replicase are inside DMVs. Because dsRNA is the first viral RNA species synthesized upon infection (*21*, *22*), these data suggest that the replicase must be rapidly enclosed inside DMVs following polyprotein translation. In contrast, newly synthesized DMV-associated RNA, metabolically labeled with 5′-ethynyl-uridine (EU) (*23*, *24*) and detected by click reaction with biotin, was partially accessible to fluorescent streptavidin (Fig. 3B and fig. S5C). Similarly, enzymatic digestion of EU-labeled viral RNA was partial in the presence of digitonin and complete with TX-100 (fig. S6, A to D). On the basis of sensitivity to ribonuclease A (RNase A), which digests ssRNA but not dsRNA (fig. S6, A and B), our data point to the newly synthesized DMV-associated RNA being single stranded. We also examined viral RNA synthesis using a run-off assay in digitonin-permeabilized cells (fig. S6E). Under these conditions, nascent DMV-associated RNA is protected from nuclease digestion (fig. S6, F and G). Together, these data indicate that, while the dsRNA intermediate and the replicase complex are inside DMVs, viral transcripts spread from inside DMVs to the cytoplasm.

We next used structural illumination microscopy (SIM) and stimulated emission depletion (STED) microscopy to pinpoint the location of key components involved in viral RNA synthesis at early stages of the viral infection [≤7 hours postinfection (hp.i.)], when the DMV pore, replicase, and dsRNA show strong colocalization (fig. S7). We focused on single DMVs (dsRNA signal diameter ≤ 250 nm) undergoing active transcription early in the infection (fig. S8, A to D), as identified by EU incorporation (Fig. 3C). Components of the pore (nsp3 and nsp4), of the replicase (nsp7, nsp8, and nsp9), and the dsRNA localized within a larger and more diffuse EU-labeled RNA signal (Fig. 3C and figs. S8I and S9, C to F). Nsp4 shows a high degree of colocalization with nsp7 and nsp8 throughout viral infection, while nsp3 colocalizes to a lesser degree (Fig. 3D and figs. S8I and S9, A and B). Using STED microscopy, we observed that the nsp4 C terminus, nsp7, nsp8, and nsp9 are always closer to the dsRNA, while the nsp3 N terminus was further away (Fig. 3, E to H, and fig. S8, E and F); this is consistent with the predicted localization of the nsp3 N terminus and nsp4 C terminus to the DMV surface and inner lumen, respectively (Fig. 1C). Furthermore, by horseradish peroxidase (HRP)–catalyzed proximity labeling, the pore, multiple replicase subunits and the dsRNA are found in each other’s vicinity (fig. S10). We note that the degree of colocalization between nsps, and between nsps and dsRNA, generally decreases as viral infection progresses (fig. S8, G and H); the reason for this is currently unknown but is consistent with the nonoverlap between replicase and dsRNA observed in past studies (*11*). Together, the results above suggest that the DMV pore and the replicase might form a complex.

**Fig. 4.**
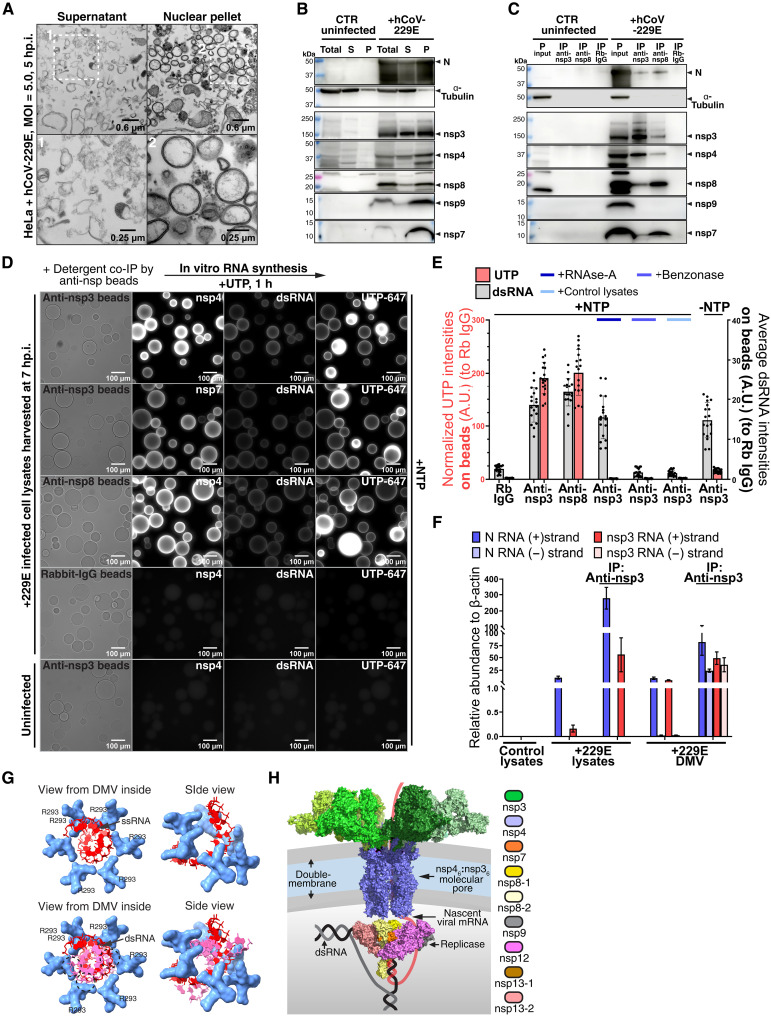
Viral RNA synthesis by a DMV replicase-pore complex. (**A** and **B**) hCoV-229E–infected cells (MOI = 5.0) were lysed, and nuclear pellets (P) or supernatants (S) were imaged by TEM (5 hp.i.) (A) or analyzed by SDS–polyacrylamide gel electrophoresis (SDS-PAGE) and immunoblotting (7 hp.i.) (B). Total, total lysates; N, nucleocapsid. (**C**) As in (B), and solubilized (P) fraction was immunoprecipitated (IP) with anti-nsp3 or anti-nsp8 antibodies followed by immunoblotting. (**D**) As in (C), but immunoprecipitates on beads were incubated with NTPs and fluorescent UTP, then fixed and stained for dsRNA and nsps. (**E**) As in (D), but showing quantification of intensities of fluorescent UTP (left *y* axis) and dsRNA (right *y* axis) on beads. Six nonoverlapping fields were analyzed per condition, and each condition was repeated thrice (*N* = 3, *n* = 18). (**F**) Positive (+) or negative (−) strand RNAs for N or nsp3 were measured by quantitative reverse transcription polymerase chain reaction. RNA abundance was normalized to β-actin mRNA in each sample. Each condition was performed in triplicate (*n* = 9). (**G**) Model of mRNA (top) and dsRNA (bottom) molecules translocating through the conserved pore constriction. The dsRNA molecule clashes sterically (dashed circles) with residues in nsp4. (**H**) Proposed model of the DMV pore-replicase complex transcribing a dsRNA template, with the nascent viral mRNA cotranslocating through the pore to the cytoplasm.

To test this hypothesis, a DMV-enriched fraction from infected cells (Fig. 4, A and B) was detergent solubilized and subjected to immunoprecipitation and immunoblotting. As shown in Fig. 4C, at 7 hp.i., nsp4, nsp7, and nsp8 are precipitated with anti-nsp3 antibodies, while nsp3, nsp4, and nsp7 are precipitated with anti-nsp8 antibodies. Furthermore, mass spectrometric analysis shows that nsp9, nsp12, and nsp13 are also coprecipitated with nsp3 and nsp8 (fig. S11, A and B). These results indicate that a pore-replicase complex exists inside DMVs. Because nsp4 is the lumen-facing pore subunit, it is likely that it is involved in contacting the replicase. We find that nsp4 interacts with nsp8 alone or with an nsp7-nsp8 complex (fig. S11E), corroborating the prediction of an nsp4-nsp8 sixfold symmetric complex by ColabFold (fig. S11F); however, whether the replicase is connected to the pore through nsp8 alone, of via a more extended molecular interface, possibly also involving RNA, remains to be determined.

Aside from the replicase, dsRNA and (−) strand viral RNAs also copurify with nsp3 (Fig. 4, D and F), and furthermore, dsRNA affinity isolated from the DMV-enriched fraction copurifies with nsp3, nsp4, nsp8, nsp9, nsp12, and nsp13 (fig. S11C). We thus tested whether the pore-replicase complex is active in RNA synthesis. When the complex immunopurified on anti-nsp3 beads is incubated with a ribonucleotide triphosphate (NTP) mix supplemented with fluorescent uridine triphosphate (UTP), a strong fluorescent signal develops on the surface of the beads (Fig. 4, D and E, and fig. S11D). This signal is dependent on the presence of the NTP mix during incubation and is sensitive to RNase A, indicating that it represents ssRNA transcribed on beads (Fig. 4E and fig. S11D). The amount of dsRNA remains constant on the beads during incubation (Fig. 4, D and E, and fig. S11D), consistent with the hypothesis that it serves as template for the RdRp, similar to how a DNA duplex is a template for RNA polymerase. Similar results were obtained when the pore-replicase complex was purified using anti-nsp8 antibody beads (fig. S11D). These results show that the pore-replicase complex is transcriptionally active, perhaps using dsRNA as template.

The conserved positively charged residues that delineate the constriction of the nsp3-nsp4 pore suggest a likely interaction with the nascent viral mRNA as it exits the DMV. Modeling predicts that the constriction can accommodate one ssRNA molecule, but it is too narrow for dsRNA (Fig. 4G). Because of these steric constraints, we hypothesized that bulky modifications of the nucleobases may interfere with mRNA passage through the constriction, leading to an antiviral effect. To test this idea, we treated cells with adenosine analogs that carry substituents of varying size on the N^6^ atom, after which we infected the cells with hCoV-229E. N^6^-benzyl-adenosine strongly inhibited viral mRNA transcription, as assayed by EU incorporation in DMVs (fig. S12, A and B), while adenosines with smaller N^6^ substituents had no effect. Similarly, N^6^-benzyl-ATP blocked run-off transcription as assayed by incorporation of fluorescent UTP in permeabilized cells, while N^6^-methyl-ATP did not (fig. S6, F and G). Inhibition of viral mRNA transcription by N^6^-benzyl-adenosine was comparable to that caused by the well-established RdRp inhibitor remdesivir (*25*, *26*) (figs. S6, F and G, and S12, A and B). While remdesivir potently blocked both dsRNA and mRNA synthesis, N^6^-benzyl-adenosine only had a modest effect on dsRNA (fig. S12, A and B), consistent with an inhibitory effect on mRNA translocation through the pore and less on the initial copying of the viral genome. All adenosine analogs competed with incorporation of the “clickable” N^6^-propargyl-adenosine into total cellular RNA (fig. S12, C and D), indicating that the analogs were properly taken up by cells, converted into NTPs and used in RNA synthesis. Last, consistent with inhibition of viral transcription, N^6^-benzyl-adenosine, but not other analogs with smaller substituents, potently blocked viral infection (fig. S12, E to G).

## DISCUSSION

Our results suggest that coronaviral DMVs share similarities with the membrane-bound replication organelles of other (+) ssRNA viruses, while also having unique characteristics (*27*–*31*). In the case of the flock house virus (FHV), the outer mitochondrial membrane spherules associated with viral replication connect to the cytoplasm via dodecameric pores (*27*, *28*). A single large protein (protein A) includes the pore-forming domain as well as an RNA capping domain and the viral RdRp; thus, the FHV pore is covalently connected to the replicase (*27*, *28*). In the case of chikungunya virus, the outward-facing plasma membrane spherules connect to the cytoplasm also via dodecameric pores. However, the nonstructural protein that forms the pore (nsP1, which is also an RNA-capping enzyme) has no transmembrane domains, presumably deforming the membrane via peripheral association. Inside the spherule, nsP1 interacts with a complex of nsP2 (an RNA helicase-protease fusion) and the nsP4 RdRp, thus forming a large pore-replicase complex (*29*, *30*). In coronaviral DMVs, we propose that a hexameric nsp3-nsp4 transmembrane protein complex forms the pore, with nsp4 playing a central role in bringing the two membranes together. The replicase inside the DMV lumen, possibly through its nsp8 subunit, interacts with the pore, most likely via the CTD of nsp4. DMVs are probably inflated by mRNA synthesis off the dsRNA template trapped inside, as implied by the larger size of viral DMVs compared to synthetic ones (*6*, *13*). Nascent viral mRNA is translocated perhaps cotranscriptionally through the pore to the cytoplasm, interacting with the highly conserved Arg motifs in nsp4 that delineate the pore constriction (fig. S13). It is unclear whether other molecules pass through the pore besides exiting mRNAs; one possibility is that all necessary macromolecules are enclosed at the time of DMV formation. The NTPs required for RNA synthesis inside the DMV might freely enter through the pore or, alternatively, might pass through as yet unidentified transporters. It appears that DMVs form rapidly after polyprotein translation and proteolytic processing, ensuring that dsRNA is immediately enclosed and shielded from innate immune detection by cytoplasmic sensors. It remains unknown how the nsp3-nsp4 pore assembles, whether and how scission of DMVs from the ER occurs, and what fusogen accomplishes the complete separation of the two concentric bilayers that limit the DMV.

Our differential accessibility data (Fig. 2) are consistent with a recent cryo-ET structure of DMV pores assembled in an artificial expression system (*40*), and they do not distinguish between it and our model (Fig. 1). In the new structure, the pore complex consists of 12 nsp3 and 12 nsp4 subunits assembled into four stacked rings, with all nsp4 subunits having their N termini in the cytoplasm and their C termini toward the inside of the DMV (*40*). The new structure also predicts a narrow central pore lined by conserved basic residues, which is very similar to our model. Thus, both models are consistent with blockade of (+) strand viral RNA (vRNA) export by bulky nucleoside analogs (fig. S12).

## MATERIALS AND METHODS

### DNA constructs

Plasmids carrying severe acute respiratory syndrome coronavirus 2 (SARS-CoV-2) and hCov-229E genes were obtained from the community depository of viral plasmids created by the Silver Lab (Department of Systems Biology, Harvard Medical School) and were used as polymerase chain reaction (PCR) templates for assembling various expression constructs. For bacterial expression, constructs were generated in the vectors pMal-c2 (NEB), pGEX-2tk (GE Healthcare) or pET30-HT7, a version of pET30 (EMD Millipore) for making N-terminal fusions to HaloTag7 (HT7, Promega). Fusions of the following proteins were expressed in bacteria: hCoV-229E N full-length, hCoV-229E nsp3 N-terminal fragment (nsp3-N, amino acids 300 to 703), hCoV-229E nsp4 CTD (nsp4-CTD, amino acids 370 to 480), hCoV-229E nsp7 full-length, hCoV-229E nsp8 full-length, hCoV-229E–nsp9 full-length, and an N-terminal fragment of human PKR (PKR-N) comprising the two dsRNA-binding domains (amino acids 1 to 181). For constitutive expression in mammalian cells, the following constructs were subcloned into the pHAGE lentiviral vector (*32*): SARS-CoV-2 nsp4 tagged with five copies of the myc epitope at the N terminus and mCherry at the C terminus (myc-nsp4-mCherry), SARS-CoV-2 nsp4 tagged with mCherry at the C terminus (nsp4-mCherry), and nuclear-localized mCherry tagged with a human protein C (HPC) epitope at the N terminus (NLS-HPC-mCherry). For tetracycline (Tet)–inducible expression in mammalian cells, a construct encoding N-terminally truncated SARS-CoV-2 nsp3 (nsp3-DN, amino acids 1224 to 1945, starting with the β-coronavirus–specific βSM domain), tagged with enhanced green fluorescent protein (eGFP) at the N terminus and HPC epitope at the C terminus, was assembled in the lentiviral vector pLIX402 (*33*).

### Cell culture

Human embryonic kidney (HEK) 293 and HeLa cells were purchased from American Type Culture Collection (ATCC). Cells were grown in Dulbecco’s modified Eagle’s medium (DMEM, Sigma-Aldrich) supplemented with 10% fetal bovine serum (Hyclone), penicillin (100 IU/ml), and streptomycin (100 μg/ml) (Thermo Fisher Scientific), at 37°C and in an atmosphere of 5% CO_2_, in a humidified incubator. HEK293 cells were transiently transfected using Mirus TransIT-293 reagent (Mirus), following the protocol from the manufacturer.

### Stable cell lines

Constructs were built in third-generation lentiviral vectors and were used to produce lentiviruses by transient transfection of HEK293 cells. Stable cell lines were then generated by lentiviral transduction for 48 hours in the presence of polybrene (1 μg/ml, Sigma-Aldrich), followed by selection with antibiotic (blasticidin, puromycin, or hygromycin), as described previously (*34*). Expression of the construct of interest was confirmed by immunofluorescence and/or Western blotting. Single clones were isolated and characterized for the stable HEK293 cells expressing Tet-inducible eGFP-nsp3-DN-HPC together with constitutively expressed nsp4-mCherry, used for assembling synthetic DMVs.

### Infection with hCoV-229E

Media containing hCoV-229E was mixed with Eagle’s minimum essential medium (EMEM; ATCC) supplemented with 2% fetal bovine serum (Hyclone). The viral mix was then added to HeLa cells (multiplicity of infection = 5.0) in a humidified incubator at 35°C and 5% CO_2_. The cells were fixed and analyzed by immunofluorescence at different times after infection, to detect viral protein expression.

### Antibodies

Antibodies against hCoV-229E nucleocapsid (N) and nonstructural proteins nsp3, nsp4, nsp7, nsp8, and nsp9 were raised in rabbits (Cocalico Biologicals) and were affinity purified against antigen covalently immobilized on beads (Affi-Gel 10 or Affi-Gel 15, Bio-Rad). Rabbit polyclonal antibodies against mCherry (*32*) and the mouse monoclonal antibody against HPC (*34*) were described before. Affinity-purified antibodies were fluorescently labeled by reaction with N-hydroxysuccinimidyl esters of various fluorophores (Alexa Fluor 488, Alexa Fluor 568, and Cy-5), according to the manufacturers’ instructions. Antibodies were separated from unincorporated fluorophores on desalting columns (NAP-5 or PD-10, GE Healthcare).

The following antibodies were obtained commercially: mouse monoclonal anti-myc 9E10 (Sigma-Aldrich), mouse monoclonal anti-dsRNA J2 (Jena Bioscience), total rabbit immunoglobulin G (IgG) (Jackson ImmunoResearch), mouse monoclonal anti–α-tubulin DM1α (Santa Cruz Biotechnology), CF488A-conjugated goat anti-rabbit secondary (Biotium), CF568-conjugated goat anti-rabbit secondary (Biotium), Alexa Fluor 594–conjugated goat anti-rabbit secondary (Thermo Fisher Scientific), Alexa Fluor 647–conjugated goat anti-mouse secondary (Thermo Fisher Scientific), and Abberior STAR RED 647–conjugated goat anti-rabbit secondary (Abberior).

### Reagents

The following reagents were purchased: Hoechst 33342 (Sigma-Aldrich), ProLong Diamond mountant (Thermo Fisher Scientific), methanol-free formaldehyde (Thermo Fisher Scientific), Tosylactivated Dynabeads (Thermo Fisher Scientific), Protein-A agarose (Sigma-Aldrich), streptavidin agarose (Thermo Fisher Scientific), digitonin (Calbiochem), EU (Cayman Chemical), actinomycin D (Cayman Chemical), biotin-azide (MedChem Express), N6-[2-(4-aminophenyl)ethyl]-adenosine (MedChem Express), N6-(4-hydroxybenzyl)-adenosine (MedChem Express), N6-benzyl-adenosine (MedChem Express), N6-methyl-adenosine (MedChem Express), N6-dimethyl-adenosine (MedChem Express), N6-propargyl-adenosine (Jena Bioscience), CF647-conjugated streptavidin (Biotium), aminoallyl–UTP–PEG_5_–Alexa Fluor 647 (Jena Bioscience), Alexa Fluor 488–NHS (*N*-hydroxysuccinimide) ester (Thermo Fisher Scientific), Alexa Fluor 568–NHS ester (Thermo Fisher Scientific), Cy5-NHS ester (MedChem Express), HaloTag succinimidyl ester (O2) ligand (Promega), HaloTag-Oregon Green ligand (Promega), HaloTag-TMR ligand (Promega), and HaloTag-biotin ligand (Promega), HRP (Thermo Fisher Scientific).

### Protein expression and purification from bacteria

Fusion proteins were expressed in BL21(DE3) pLysS *Escherichia coli* (EMD Millipore). The proteins were tagged at the N terminus with his_6_-HT7 (pET30-HT7 vector), maltose-binding protein (MBP) (pMal-c2 vector) or glutathione *S*-transferase (GST) (pGEX-2tk vector). Bacterial pellets were lysed in lysis buffer [50 mM tris (pH 7.5), 150 mM NaCl, 1% TX-100, and 1 mM phenylmethylsulfonyl fluoride]. In the case of his_6_-HT7 fusions, the lysis buffer was supplemented with 25 mM imidazole (pH 7.5). Lysates were clarified by centrifugation, and the recombinant protein was purified according to the manufacturers’ instructions, using Ni–nitrilotriacetic acid agarose (Qiagen), amylose resin (NEB), or glutathione agarose (GE Healthcare). Eluted protein was dialyzed against Hepes-buffered saline [HBS; 10 mM Na-Hepes (pH 7.5) and 150 mM NaCl] overnight at 4°C, concentrated using Amicon centrifugal filters (Sigma-Aldrich), frozen in liquid nitrogen, and stored at −80°C. The probe for dsRNA detection consisted of his_6_HT7-tagged hPKR-N. The following proteins were used for rabbit immunizations: MBP-229E-N, MBP-229E-nsp3-N, GST-229E-nsp4-CTD, his_6_HT7-229E-nsp7, MBP-229E-nsp8, and his_6_HT7-229E-nsp9. The following proteins were used to generate affinity matrices for antibody purification from immune sera: GST-229E-N, GST-229E-nsp3-N, GST-229E-nsp4-CTD, his_6_HT7-229E-nsp7, his_6_HT7-229E-nsp8, and MBP-229E-nsp9.

### Probes for detecting dsRNA

Viral dsRNA was detected using purified recombinant his_6_Halo-hPKR-N labeled with a fluorophore, biotin, or HRP. Fluorescently and biotin-labeled his_6_Halo-hPKR-N were generated by incubating the recombinant protein with HaloTag-fluorophore or HaloTag-biotin ligands, according to the manufacturer’s instructions. Excess HaloTag ligand was then removed by purification on desalting NAP-5 or PD-10 columns (GE Healthcare). To generate the HRP-his_6_Halo-hPKR-N conjugate, purified HRP was first modified with HaloTag succinimidyl ester (O2) ligand and was then reacted with purified his_6_Halo-hPKR-N; we estimated that a 1:1 molar ratio between the proteins resulted in >90% modification of his_6_Halo-hPKR-N. An immunofluorescence protocol (see below) was used to stain fixed virus-infected cells with various dsRNA probes.

### Immunofluorescence

HEK293 cells expressing genes of interest or HeLa cells infected with hCoV-229E were fixed with 2% formaldehyde in phosphate-buffered saline (PBS), for 10 min at room temperature. After washing with tris-buffered saline (TBS), the cells were permeabilized with 40 μM digitonin (3 to 5 min) or 0.2% TX-100 (10 min) in TBS. Cells were counterstained with Hoechst 33342 (1 μg/ml) and then blocked with blocking solution (3% bovine serum albumin in PBS). For indirect immunofluorescence, the cells were incubated with unlabeled primary antibodies diluted in blocking solution, overnight at 4°C. After washing with PBS, the cells were incubated with labeled secondary antibodies in blocking buffer, for 1 hour at room temperature. The cells were washed in PBS, were mounted in mounting media (Prolong Diamond), and were imaged by fluorescence microscopy. For direct immunofluorescence, cells were stained with labeled primary antibodies diluted in blocking buffer, overnight at 4°C. For detection of dsRNA, virus-infected cells were incubated with fluorescently conjugated his_6_Halo-hPKR-N diluted in blocking buffer, for 15 min at room temperature. For the proteinase K accessibility assay, HEK293 cells coexpressing nsp3 and nsp4 were permeabilized with 40 μM digitonin (3 to 5 min) in PBS buffer. The cells were then washed once with PBS, followed by incubation with proteinase K (10 μg/ml) in PBS. After the indicated time, the cells were fixed with 3.7% formaldehyde in PBS and were processed for fluorescence microscopy.

### Detection of viral RNA synthesis in cells by click chemistry

hCoV-229E–infected HeLa cells were preincubated with actinomycin D (10 μM) for 1 hour, to suppress host cell transcription. The cells were then incubated with EU (1 mM) for 45 min in the continued presence of actinomycin D, followed by fixation with 2% formaldehyde in PBS for 15 min at room temperature. The cells were permeabilized with either 40 μM digitonin or 0.2% TX-100 in TBS, washed with TBS, and then stained with biotin-azide (10 μM) in click buffer [100 mM tris (pH 8.5), 1 mM CuSO_4_, and 100 mM ascorbic acid] for 30 min at room temperature. The cells were washed with TBS and stained with fluorescent streptavidin conjugates diluted in blocking buffer, followed by fluorescence microscopy. For testing differential digestion of viral RNA by nucleases, EU-labeled cells fixed and permeabilized as above were incubated with RNase A (4 ng/μl) or benzonase (5 U/μl) in 1× CutSmart buffer (NEB), for 1 hour at 37°C. Cells were washed thoroughly with TBS, and incorporated EU was detected by click reaction as above.

### Immunoprecipitation

To immunopurify DMVs, virus-infected HeLa cells were resuspended in hypotonic lysis buffer [10 mM Na-Hepes (pH 7.6), 10 mM Na acetate, and 1.5 mM MgCl_2_] and were lysed on ice using a Dounce homogenizer (30 strokes). The lysate was centrifuged for 10 min at 650*g* and 4°C; the nuclear pellet was resuspended in HBS and was incubated with anti-nsp3 antibodies immobilized on magnetic beads, for 2 hours at 4°C. The beads were washed three times with HBS, and precipitated proteins or RNAs were analyzed by immunoblotting or quantitative reverse transcription PCR (RT-PCR), respectively. To immunoprecipitate viral proteins, virus-infected HeLa cells or nuclear pellets thereof (see above) were lysed in HBS with 1% TX-100 and protease inhibitors. The lysate was clarified by centrifugation for 20 min at 14,000*g* and 4°C and was incubated with anti-nsp3, anti-nsp8, or total rabbit IgG (negative control) antibodies immobilized on magnetic beads, for 2 hours at 4°C. The beads were washed three times with HBS with 1% TX-100, and copurified viral proteins and RNAs were analyzed as above for immunopurified DMVs.

### Protein electrophoresis and immunoblotting

Protein samples were mixed with SDS sample buffer supplemented with 50 mM dithiothreitol (DTT), boiled, and then subjected to SDS–polyacrylamide gel electrophoresis (SDS-PAGE) (4 to 20% Criterion TGX Precast Midi Gels, Bio-Rad). Separated proteins were visualized by staining with Coomassie Blue R250 (G-Biosciences). Alternatively, the separated proteins were transferred to polyvinylidene difluoride membranes (Bio-Rad), which were rinsed with TBS with 0.1% Tween 20 (TBST), blocked with 5% nonfat milk in TBST, and incubated with primary antibodies in TBST with milk, overnight at 4°C. The membrane was washed with TBST and was incubated with HRP-conjugated secondary antibodies in TBST with milk, for 1 hour at room temperature. The membrane was washed again with TBST, incubated for 1 min with chemiluminescent substrate (Revvity), and then imaged (Amersham ImageQuant 800, Cytiva).

### Isolation of synthetic DMVs by sucrose gradient centrifugation

Sucrose gradients (20 to 60%) in centrifugation buffer [10 mM Na-Hepes (pH 7.6) and 75 mM NaCl] were prepared in 14 mm by 95 mm polypropylene tube (Beckman), using Gradient Master (BIOCOMP). HEK293 cells expressing synthetic DMVs were harvested and resuspended in ice-cold hypotonic lysis buffer. The cells were lysed in a Dounce homogenizer on ice, with 15 strokes. Lysates were centrifuged for 15 min at 500*g* and 4°C. The supernatant was collected, adjusted to 75 mM NaCl and 5% sucrose, and was layered on top of the sucrose gradient. The sucrose gradients were centrifuged for 4 hours at 36000 rpm and 4°C, in an SW-40 rotor (Beckman). Gradient fractions were collected using a gradient fractionation system (BRANDEL) and were analyzed by SDS-PAGE and immunoblotting. Fractions enriched in eGFP-ΔN-nsp3-HPC and nsp4-mCherry were pooled and subjected to immunoprecipitation with anti-HPC antibody covalently immobilized on magnetic beads (Tosylactivated Dynabeads, Thermo Fisher Scientific). The beads were washed three times with immunoprecipitation buffer and fixed by a fixation buffer (described in the EM method section) for electron microscopy analysis.

### Proximity labeling assay and denaturing coimmunoprecipitation

HeLa cells infected with hCoV-229E were fixed with 1% formaldehyde in PBS, for 10 min at room temperature. Cells were washed in PBS, permeabilized, and incubated with rabbit primary antibodies against nsp4, nsp7, or nsp8, followed by HRP-conjugated goat anti-rabbit secondary antibody. After washing with PBS, cells were incubated with biotin-tyramide (5 μM, MiliporeSigma) in HRP staining buffer [100 mM tris (pH 7.5), 150 mM NaCl, 0.1% Tween 20, and 0.003% H_2_O_2_], for 15 min at room temperature. Cells were washed with TBS, collected, and incubated for 1 hour at 65°C in 50 mM tris (pH 7.5), 150 mM NaCl, and 2.5% SDS. The samples were centrifuged for 15 min at 14,000*g*; the supernatant was diluted with TBS to 0.5% SDS, and was incubated with streptavidin agarose for 2 hours at room temperature. The beads were washed with TBS + 0.5% SDS, and the bound protein was eluted with 62.5 mM tris (pH 6.8), 2% SDS, 10% glycerol, and bromophenol blue (1 mg/ml) at 65°C for 15 min. Eluted material was analyzed by SDS-PAGE and immunoblotting.

### Run-off viral RNA synthesis

Virus-infected HeLa cells were rinsed in ice-cold PBS, incubated with 40 μM digitonin for 3 to 5 min on ice, and then washed thoroughly with run-off buffer [10 mM K-Hepes (pH 7.6), 50 mM KCl, 2 mM MgCl_2_, 200 mM sucrose, and protease inhibitor cocktail]. The cells were incubated afterwards with run-off buffer supplemented with NTPs (250 μM each) and aminoallyl–UTP–PEG_5_–Alexa Fluor 647 (50 μM), for 1 hour at 35°C. The cells were then fixed, washed, and imaged by fluorescence microscopy.

### Viral RNA synthesis on beads

Nuclear pellets from hCoV-229E–infected HeLa cells lysed in hypotonic solution were solubilized in TBS with 1% TX-100 and were incubated with rabbit anti-nsp3, anti-nsp8, or total IgG (negative control) antibodies immobilized on magnetic beads. After washing, the beads were incubated with PBS supplemented with 0.02% TX-100, 2 mM MgCl_2_, 250 μM of each NTP, and 100 μM aminoallyl–UTP–PEG_5_–Alexa Fluor 647, for 1 hour at 35°C. The beads were washed and imaged by fluorescence microscopy.

### Quantitative RT-PCR

Total RNA coimmunoprecipitated with viral proteins was isolated using TRIzol (Thermo Fisher Scientific), according to the manufacturer’s protocol. Purified RNA was reverse transcribed with Luna Superscript Mix (NEB). RNA transcripts of viral and host genes were measured by quantitative PCR using primers listed in table S1 and Power SYBR Green kit (Thermo Fisher Scientific), on a QuantStudio7 Pro Real-time PCR System (Thermo Fisher Scientific).

### Mass spectrometric analysis

Coimmunoprecipitated proteins were eluted from beads with SDS sample buffer [62.5 mM tris (pH 6.8), 2% SDS, 10% glycerol, and bromophenol blue (1 mg/ml)], for 15 min at room temperature. The eluate was supplemented with DTT to 50 mM and was incubated for 10 min at 60°C. The samples were loaded onto a 4 to 20% Criterion TGX Precast Midi Gel (Bio-Rad) and were separated until the dye front migrated ~1.5 cm. The gel was fixed in 50% ethanol and 10% acetic acid for 30 min and was stained with Coomassie Blue R250 (G-Biosciences). The portions of the gel containing the samples were excised, and the gel pieces were subjected to in-gel trypsin digestion (*35*). Tryptic peptides extracted from the gel were then dried and stored at 4°C until mass spectrometric analysis. The peptide samples were separated on a nano-scale reverse-phase high-performance liquid chromatography capillary column (100-μm inner diameter and 30-cm length) packed with 2.6-μm-diameter spherical C18 silica beads, using a linear gradient of acetonitrile (*36*). Eluting peptides were subjected to electrospray ionization and then introduced into an Orbitrap Exploris 480 mass spectrometer (Thermo Fisher Scientific). Peptides were detected, isolated, and fragmented, and the resulting fragmentation pattern was used to determine peptide sequences using Sequest software (Thermo Fisher Scientific) (*37*).

### Microscopic imaging

Cells were grown on glass coverslips (no. 1.5, Electron Microscopy Sciences), were processed for immunofluorescence, and were imaged by spinning-disc confocal microscopy, three-dimensional–structural illumination microscopy (3D-SIM), or STED microscopy. Unless otherwise specified, all individual imaging experiments were repeated at least three times on three different days (*N* = 3, *n* = 9).

Spinning-disk confocal imaging was performed on a Nikon Ti-E inverted microscope equipped with a Yokagawa CSU-X1 spinning disk unit and ORCA-Fusion BT scientific complementary metal-oxide semiconductor (sCMOS) camera and controlled with the Nikon NIS-Elements software. Images were acquired using either a Nikon PlanFluor 40× 1.30 numerical aperture (NA) oil objective or a Nikon PlanApo 100× 1.45 NA oil objective. For deconvolution of confocal image stacks acquired with the 100× objective, a theoretical point spread function was generated with the PSF Generator plugin using the Born & Wolf 3D Optical Model, with a voxel depth of 0.3 μm and 1.45 NA. Afterwards, the deconvolution was done with the DeconvolutionLab2 plugin using a Richardson-Lucy algorithm with 10 iterations.

3D-SIM imaging was performed on an OMX V4 Blaze microscope (GE Healthcare) equipped with three PCO.edge sCMOS cameras and 488- and 568-nm laser lines. Images were acquired with a 0.125-mm step size without binning, using a 60× 1.42 NA PlanApochromat objective (Olympus). For each z-section, 15 raw images (three rotations with five phases each) were acquired. Spherical aberration was minimized using immersion oil matching (*38*). Super-resolution images were computationally reconstructed from the raw datasets with a channel-specific, measured optical transfer function and a Wiener filter constant of 0.001, using CUDA-accelerated 3D-SIM reconstruction code based on the study by Gustafsson *et al.* (*39*). TetraSpeck beads (Thermo Fisher Scientific) or a nanogrid control slide (GE Healthcare) were used to measure axial and lateral chromatic misregistration, and experimental datasets were registered using the imwarp function in MATLAB (MathWorks).

STED imaging was performed on an Abberior Instruments Expert Line system working in a time-gated configuration, implemented on an inverted Olympus IX83 microscope. Images were acquired with an Olympus UPlanSApo 100× 1.4 NA objective using 1.518 RI immersion oil. Alexa Fluor 594 and Abberior STAR RED signals were acquired by using 561- and 640-nm laser lines for excitation, respectively. For both channels, we used a 775-nm STED beam for depletion. For the Alexa Fluor 594 channel, fluorescence was detected in the range of 595 to 665 nm, and for the Aberrior STAR RED channel in the 650- to 750-nm range. For a region of interest, Z-stacks were acquired for a range of a few micrometers (depending on the size of the imaged structures), using a z-step of 100 nm. For deconvolution purposes, xy scanning was oversampled by using a 10-nm pixel size. STED images were deconvolved with Huygens Professional software (SVI, The Netherlands), using the express wizard operating in standard profile. For deconvolution parameters, a STED saturation factor of 30 and a STED immunity fraction of 10% were used.

### Image analysis

For quantifying the number and intensity of nsp and dsRNA puncta, images were analyzed using the “analyze particle” function in ImageJ. All images acquired for an experiment were subjected to the same thresholding. For profiling the signal intensity distribution of puncta in an image, mean intensities were assigned to 300 bins in all experimental groups. Fluorescent intensity line scans were generated using the “plot profile” function, and line scans of different channels were merged into one graph. Colocalization analysis was performed using the coloc-2 plugin, and a Pearson coefficient was used assess the degree of colocalization. Three nonoverlapping fields of view were analyzed for each experimental condition, and each condition was repeated three times, on different days.

### Electron microscopy

For visualizing DMVs in cells, HEK293 cells expressing synthetic DMVs or HeLa cells infected with hCoV-229E were grown as monolayers on Aclar coverslips (Electron Microscopy Science). The cells were fixed with 1.25% formaldehyde, 2.5% glutaraldehyde, and 0.03% picric acid in 0.1 M (pH 7.4) sodium cacodylate buffer, for 2 hours at room temperature. The coverslips were then washed in cacodylate buffer and were postfixed in 1% osmium tetroxide/1.5% potassium ferrocyanide for 1 hour, followed by washes with water and 50 mM maleate buffer (pH 5.15) (MB). After incubation with 1% uranyl acetate in MB for 1 hour, the cells were washed with MB and water, followed by stepwise dehydration to 100% ethanol. After carefully removing the ethanol, the cells were layered with a drop of TAAB Epon (TAAB Laboratories Equipment Ltd) and covered with another sheet of Aclar, followed by polymerization at 60°C for 48 hours. The top Aclar was then peeled off, and a small area (~1 mm) of the embedded cells was cut out with a razor blade and remounted on an Epon block. Ultrathin sections (80 nm) were cut on a Reichert Ultracut-S microtome, picked up on copper grids stained with lead citrate, and imaged on a Tecnai G2 Spirit BioTWIN transmission electron microscope equipped with an AMT NanoSprint43-MKII (43 megapixel) charge-coupled device camera.

### Data quantification and statistical analysis

Statistical information for each experiment is provided in the figure legends. For all image analysis, at least three nonoverlapping fields of view were acquired per condition, and each experiment was repeated at least three times, on different days. All biochemistry experiments (including synthetic DMV isolation and detection, immunoprecipitation of viral proteins, dsRNA affinity isolation, proximity labeling, quantitative PCR, and in vitro viral RNA synthesis) were repeated independently at least three times. Mass spectrometric and electron microscopy experiments were performed at least twice, on different days.
