# Supplementary material for: A coronaviral pore-replicase complex links RNA synthesis and export from double-membrane vesicles
Source: Sci Adv. 2024 Nov 8;10(45):eadq9580. doi: 10.1126/sciadv.adq9580 (PMC11546809; doi:10.1126/sciadv.adq9580)
Supplement: Supplementary file 1 — Figs. S1 to S15 Table S1 [file sciadv.adq9580_sm.pdf]

Science Advances
AAAS

Supplementary Materials for

A coronaviral pore-replicase complex links RNA synthesis and export from double-membrane vesicles

Anan Chen *et al.*

Corresponding author: Timothy J. Mitchison, timothy_mitchison@hms.harvard.edu;
Adrian Salic, asalic@hms.harvard.edu

Sci. Adv. **10**, eadq9580 (2024)
DOI: 10.1126/sciadv.adq9580

This PDF file includes:

Figs. S1 to S15
Table S1

Figure S1.

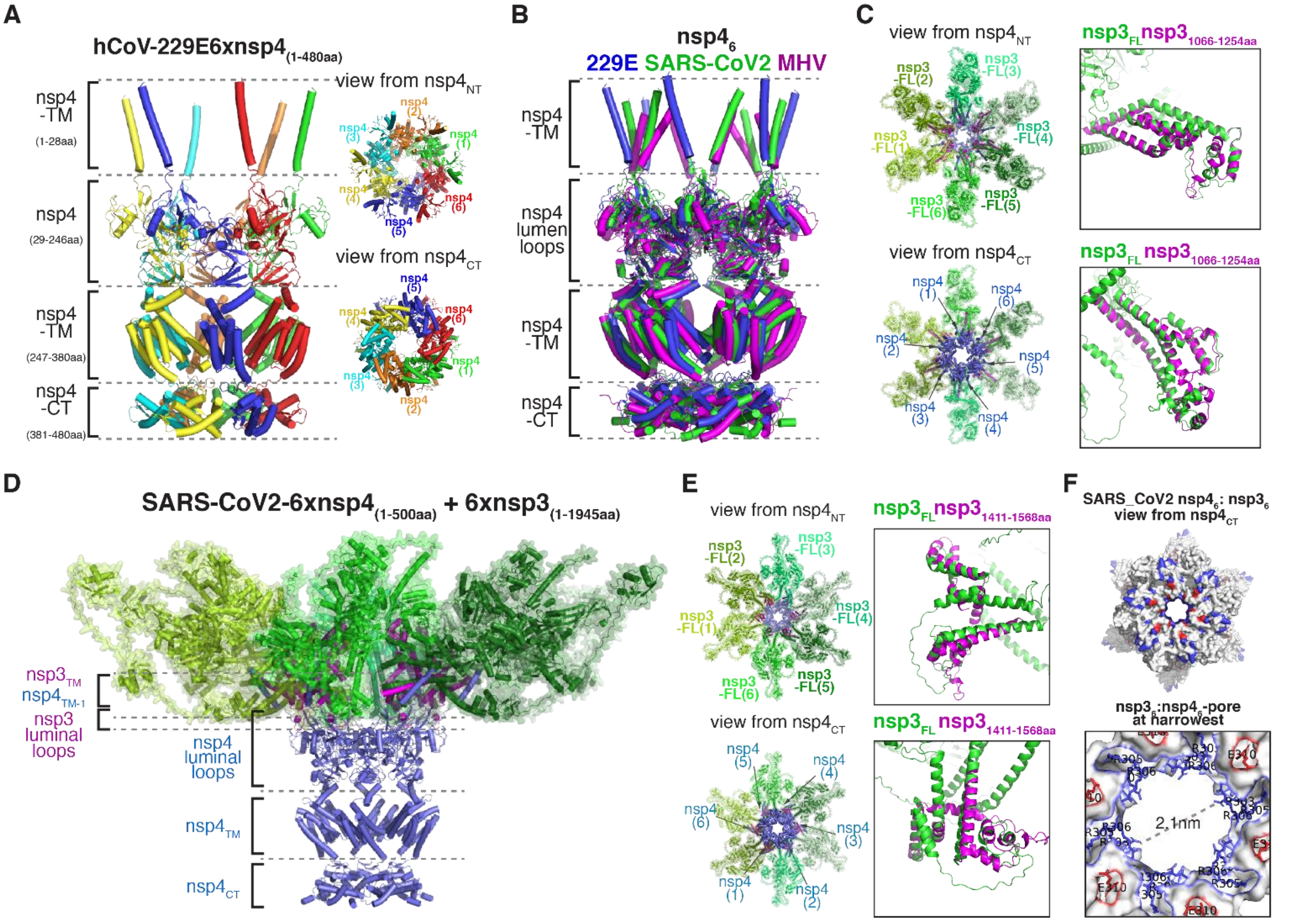

Fig. S1. Predicted nsp3-nsp4 and replicase subunits-nsp4 complexes

(A) Predicted structure of a six-fold symmetric complex of nsp4 from hCoV-229E, seen from the side (left) and from the top and bottom (right). TM = transmembrane domains; NT = N-terminus; CT = C-terminus. **(B)** As in (A), but including hCoV-229E (blue), SARS-CoV2 (green), and MHV (purple). **(C)** Predicted structure of a six-fold symmetric complex between hCoV-229E nsp3 (residues 1066-1254) and full-length nsp4. Left: top and bottom views of ribbon diagrams; right: close-up view of the alignment between hCoV-229E full length nsp3 (green) and nsp3 (1066-1254aa) (purple). **(D)** Predicted structure of a six-fold symmetric complex between SARS-CoV2-nsp3 (residues 1411-1568aa) and full-length nsp4, including the predicted structure of full-length SARS-CoV2-nsp3, seen from the side. **(E)** As in (D), but showing top and bottom views of the complex, respectively. Left: ribbon diagram; right: close-up view of the alignment between SARS-CoV2 full length nsp3 (green) and nsp3 (1411-1568aa) (purple). **(F)** As in (D), Top: molecular surface showing positive (blue) and negative (red) residues viewing from $nsp4_{CT}$; bottom: predicted narrowest portion of the pore formed by SARS-CoV2 nsp4 in complex with nsp3. The diameter of the constriction and the conserved residues delineating it are indicated.

Figure S2.

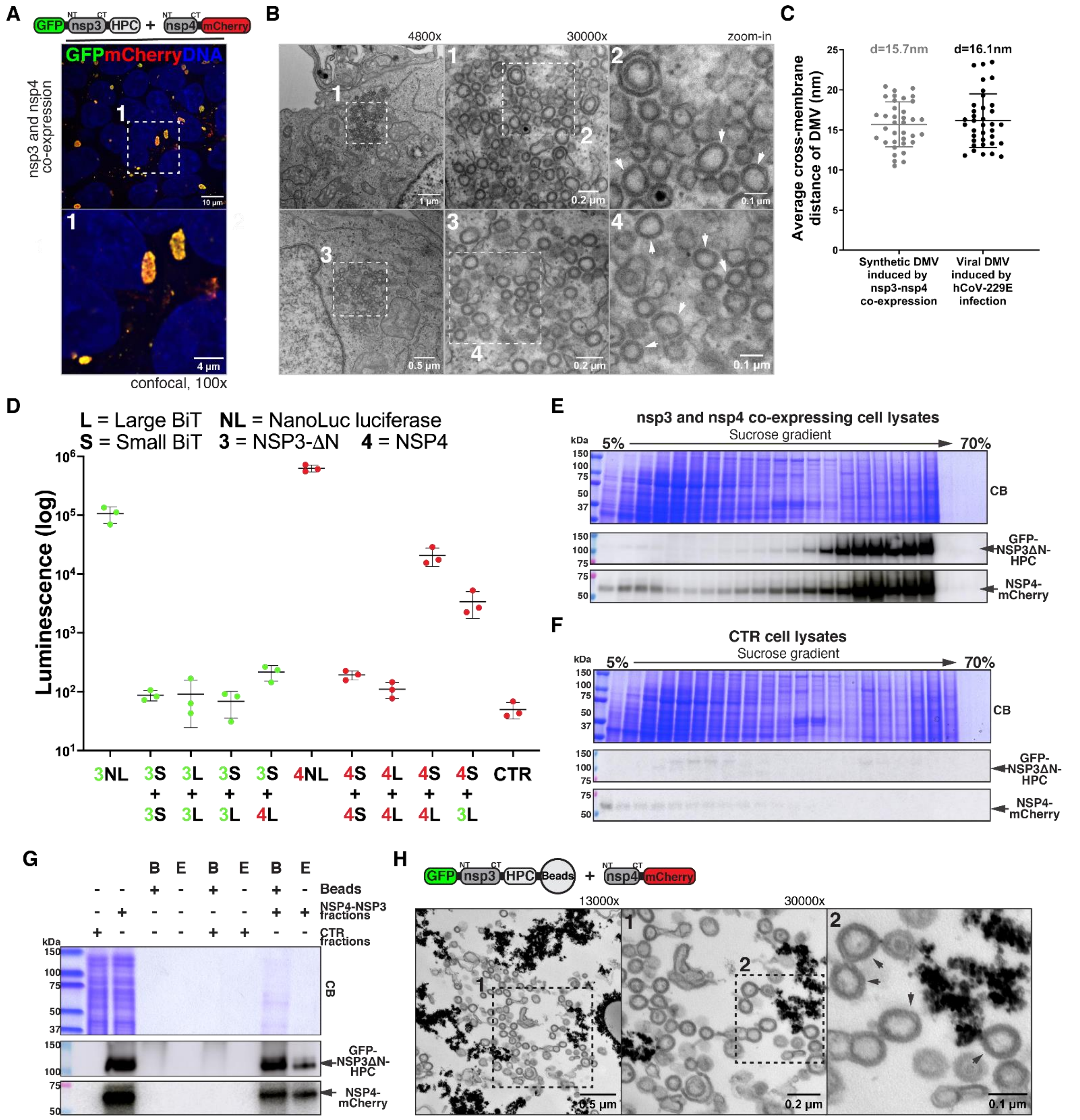

Fig. S2. DMV reconstitution by nsp3-nsp4 co-expression and DMV affinity isolation

(A) Fixed HEK293 cells expressing eGFP-nsp3ΔN-HPC and nsp4-mCherry were imaged by confocal microscopy (100×). Nsp3 and nsp4 colocalize. **(B)** As in (A), but cells were imaged by transmission electron microscopy (TEM). Middle panel: DMV-enriched cellular regions were imaged at 30000×. Right panel: zoom-in of the ROI from the middle panel, showing the double membrane of individual DMVs (white arrows). **(C)** Quantification of intermembrane distance for DMVs induced by nsp3-nsp4 co-expression or by hCoV-229E infection. A total of 36 DMVs were measured for each group (n=36). **(D)** Cells expressing nsp3-NanoLuc (NL), nsp4-NanoLuc (NL), or co-expressing a combination (as indicated in the graph) from the following constructs: nsp3ΔN-LargeBit (3L), nsp3ΔN-SmallBit(3S), nsp4-LargeBit(4L), nsp4-SmallBit(4S), were lysed and assayed for luciferase activity. Robust luciferase activity is observed when 3S and 4L, or 4S and 4L are co-expressed, indicating nsp3 interacts with nsp4 and nsp4 interacts with itself. **(E)** HEK293 cells co-expressing GFP-nsp3ΔN-HPC and nsp4-mCherry were subjected to hypotonic lysis (detergent-free), and lysates were separated by ultra-centrifugation on a sucrose gradient (5%-70%). Gradient fractions were analyzed by SDS-PAGE and immunoblotting with anti-HPC or anti-mCherry antibodies. Nsp3 and nsp4 are enriched in the same gradient fractions. **(F)** As in (E), but using control HEK293 cells. **(G)** As in (E), but nsp3- and nsp4-enriched gradient fractions were subjected to immunoprecipitation using anti-HPC antibodies. Precipitated material was eluted from beads with HPC peptide. Material on beads (B) and in eluate (E) was analyzed by SDS-PAGE and immunoblotting with anti-HPC or anti-mCherry antibodies. **(H)** As in (G), but the beads were imaged by TEM (magnification 13000×, left; 30000×, right). Synthetic DMVs (black arrows) are immunoprecipitated on the beads.

Figure S3.

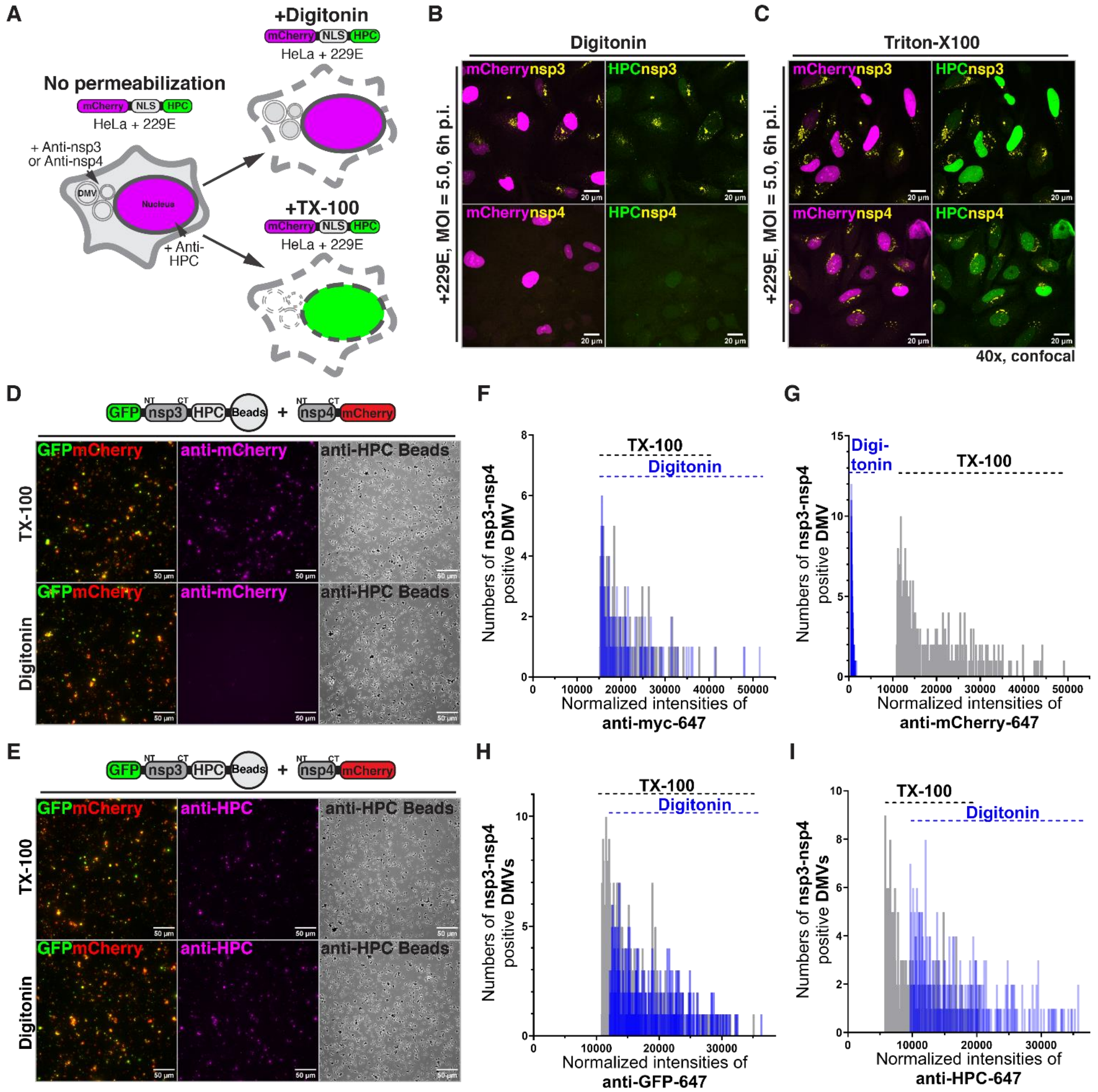

Fig. S3. Differential accessibility of assays for nsp3 and nsp4

(A) Schematic of differential antibody accessibility assay. HeLa cells expressing HPC-tagged NLS-mCherry were infected with hCoV-229E (MOI = 5.0, 6hp.i.). Cells were fixed, differentially permeabilized and stained with fluorescent anti-HPC antibodies. **(B)** As in (A), but cells permeabilized with digitonin and co-stained with anti-nsp3-NT and anti-HPC antibodies (top panel) or with anti-nsp4-CT and anti-HPC antibodies (bottom panel). Only the cytoplasm-facing nsp3-NT is detected by antibodies. **(C)** As in (B), but with TX-100 permeabilization. HPC-NLS-mCherry, nsp3 and nsp4 are all detected. **(D)** Synthetic DMV were isolated from HEK293 cells co-expressing eGFP-nsp3ΔN-HPC and nsp4-mCherry on anti-HPC beads (described in Fig. S2C-F). The beads were fixed, differentially permeabilized and stained with anti-mCherry antibodies. The mCherry tag is detected with TX-100 but not with digitonin. **(E)** As in (D), but with anti-HPC antibodies. The HPC tag nsp3 C-terminus is detected with both digitonin and TX-100. **(F-I)** Fixed HEK293 cells co-expressing eGFP-nsp3ΔN-HPC and myc-nsp4-mCherry were differentially permeabilized and stained with indicated antibodies. Measured DMV intensities were assigned into 300 bins and the number of DMVs in each bin were quantified. EGFP-, HPC-, or myc-positive DMVs have similar intensity profile between digitonin (blue) and TX-100 (grey) permeabilization, while mCherry-positive DMVs have much weaker intensity profile with digitonin.

Figure S4.

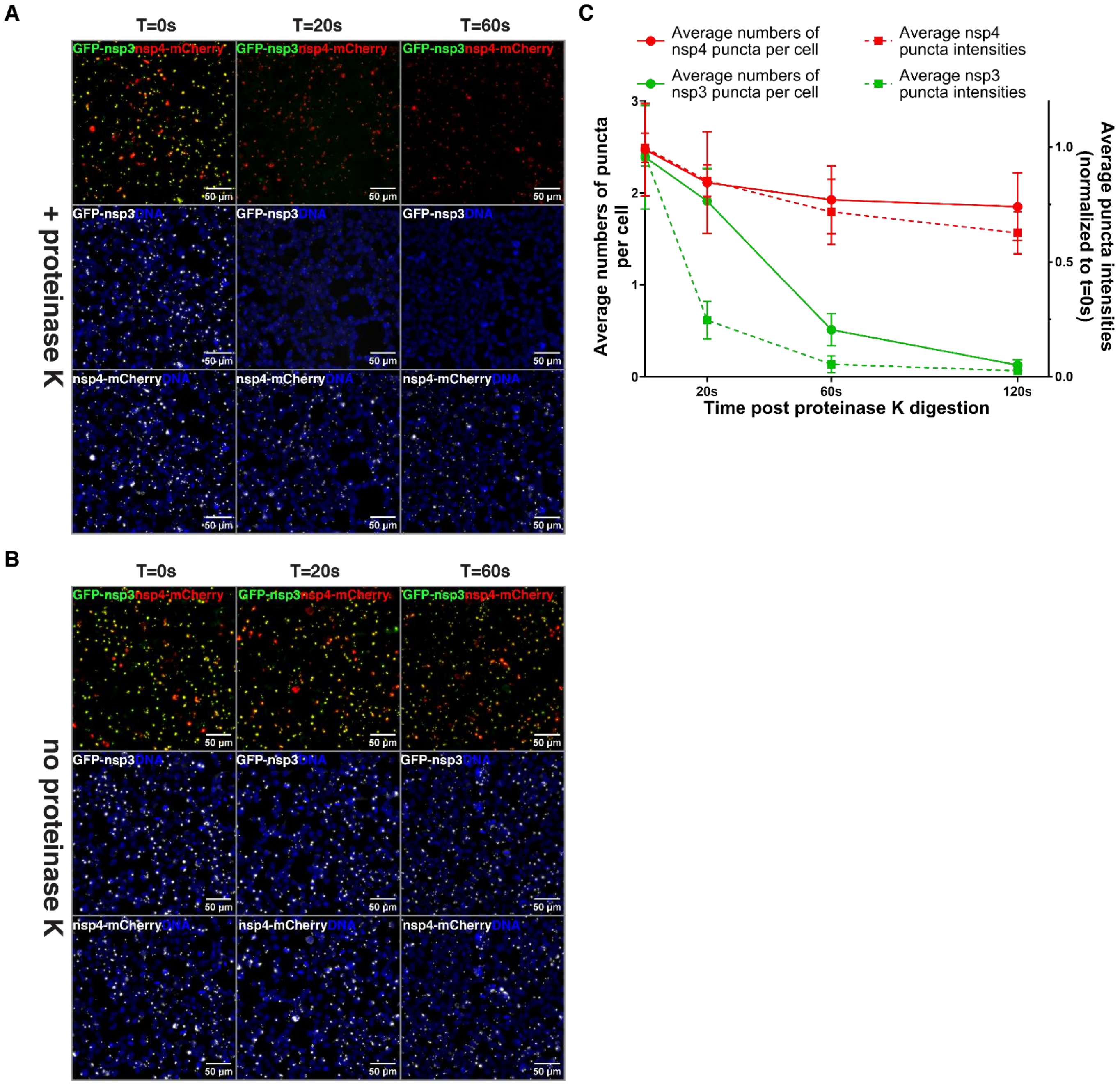

Fig. S4. Proteinase K digests nsp3 but not nsp4 under digitonin permeabilization conditions

(A) HEK293 cells co-expressing eGFP-nsp3ΔN-HPC and myc-nsp4-mCherry were permeabilized with digitonin, followed by incubation with proteinase K. Cells were fixed after different incubation times and were analyzed by immunofluorescence. The GFP signal disappears quickly, while the mCherry signal persists. **(B)** As in (A), but cells were incubated in the absence of proteinase K. Both GFP and mCherry signals are stable. **(C)** Average intensity and number of GFP and mCherry puncta were measured for the experiments in (A) and (B). At least three fields of view were analyzed for each condition, and each condition was repeated 3 times (*N*=3, n=9).

Figure S5.

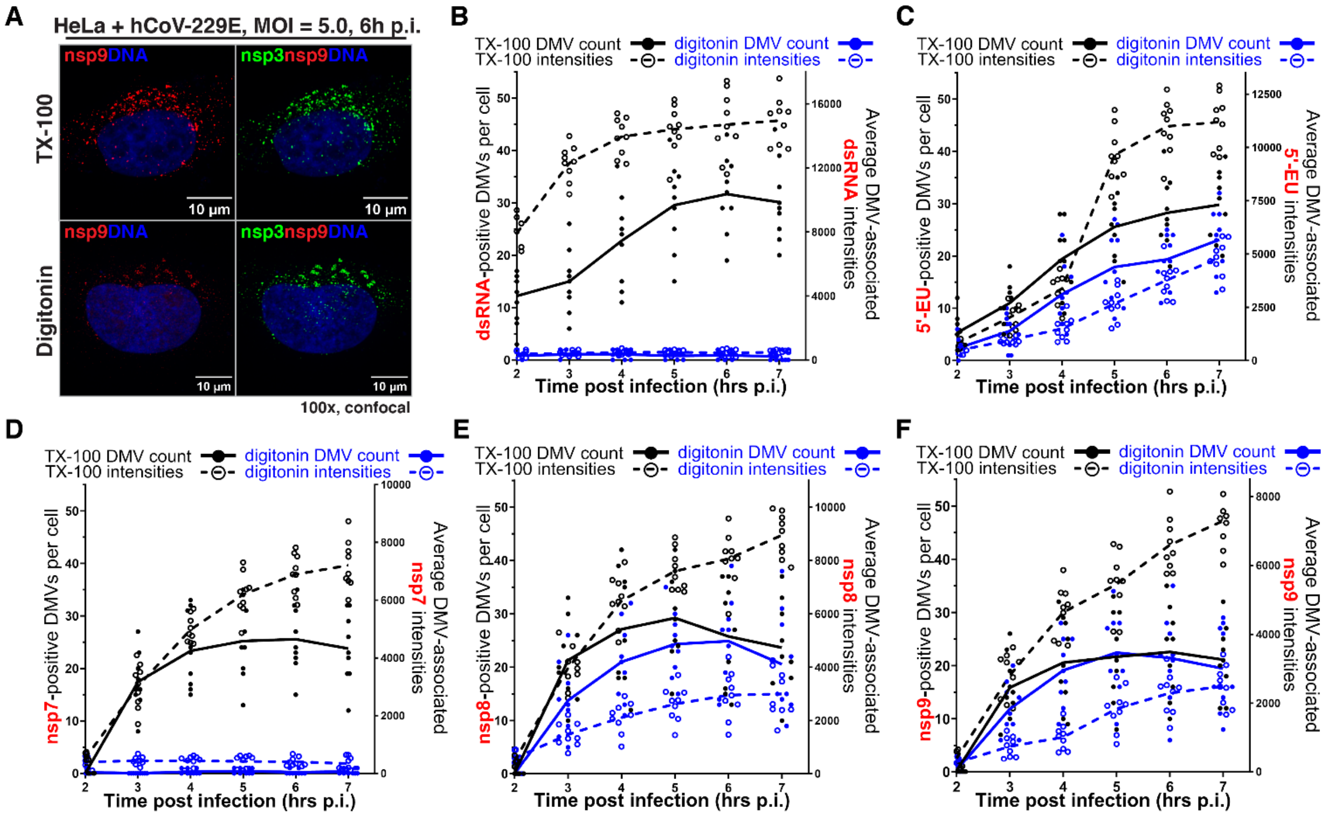

Fig. S5. Replicase subunits and nsp4 localize inside DMVs at early infection stages

(A) hCoV-229E-infected HeLa cells (MOI = 5.0, 6hp.i.) were assayed for differential accessibility using a mix of fluorescently labeled anti-nsp3 and anti-nsp9 antibodies. Nsp9 is weakly accessible with digitonin permeabilization, in contrast to nsp3. **(B-F)** hCoV-229E-infected HeLa cells were assayed for differential accessibility using the dsRNA probe (B), or with click chemistry detection of EU incorporated at DMVs (C), or anti-nsp7 antibodies (D), or anti-nsp8 antibodies (E), or anti-nsp9 antibodies (F) at different post infection time points. Three non-overlapping fields of view were analyzed per condition and each condition was repeated 3 times. (*N*=3, n=9).

Figure S6.

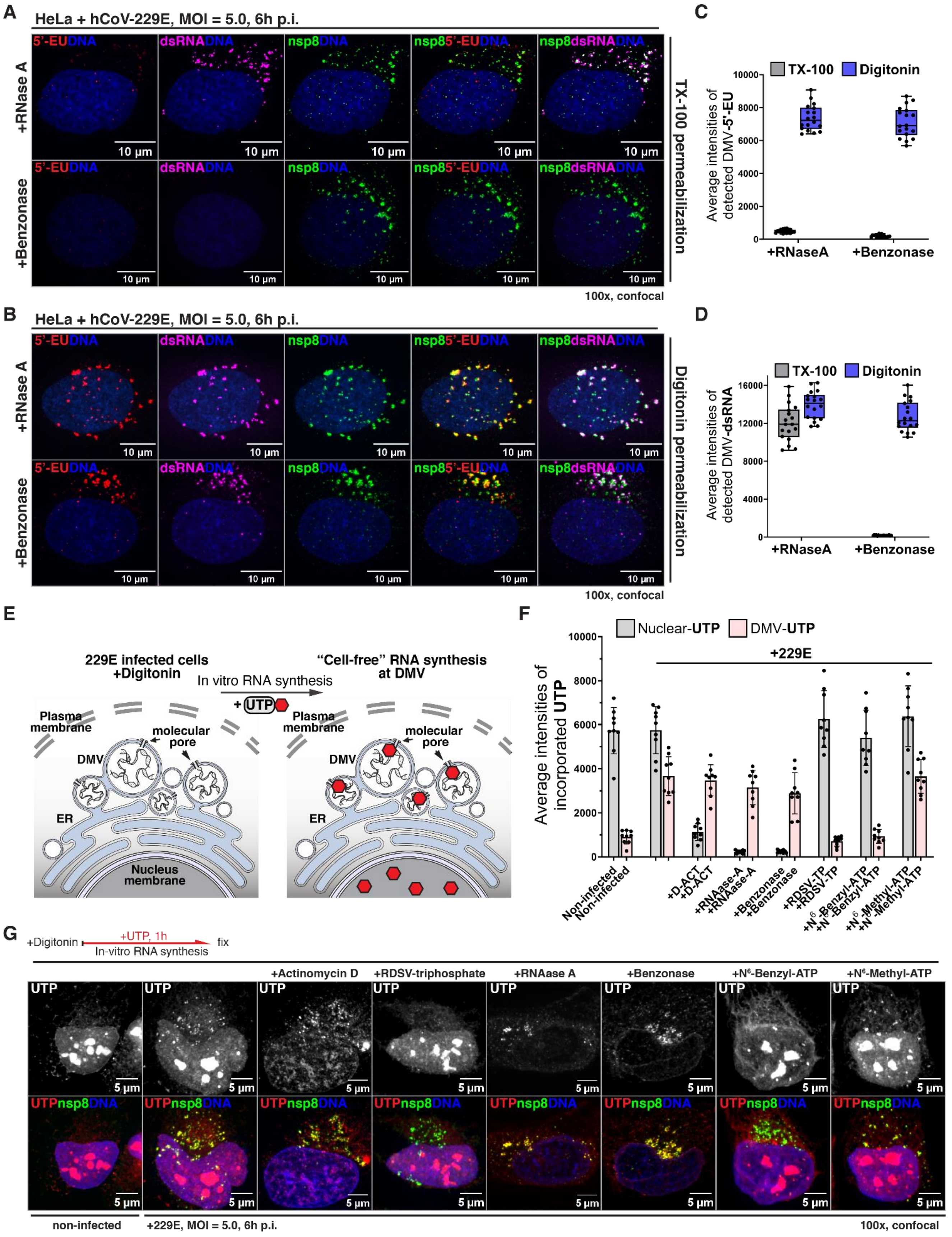

Fig. S6. Nascent viral mRNA is synthesized in DMVs and then spreads to cytoplasm

(A and B) hCoV-229E-infected HeLa cells (MOI = 5.0, 6hp.i.) were labeled with EU, fixed and permeabilized by TX-100 (A) or digitonin (B). The cells were digested with enzymes and reacted by click reaction with biotin-azide, followed by staining with fluorescent streptavidin, dsRNA probe and anti-nsp8 antibodies. **(C and D)** Quantification of average EU or dsRNA intensities at DMV under different conditions in (A and B). EU-labeled RNA is digested by RNase A with TX-100, but resistant to RNase A with digitonin permeabilization. DsRNA is only digested by benzonase with TX-100 permeabilization. Thus, nascent viral mRNA spreads from inside DMVs to the cytoplasm, but dsRNA resides strictly inside DMVs. **(E)** Schematic of run-off RNA synthesis assays. hCoV-229E-infected HeLa cells (MOI = 5.0, 6hp.i.) were permeabilized with digitonin and incubated with a mix of NTPs and fluorescent UTP. Cells were then fixed and stained with anti-nsp8 antibodies. **(F and G)** As in (E), but RNA synthesis was performed in the presence of indicated compounds or enzymes. Fluorescent UTP intensity in DMVs and nucleus was measured, in three non-overlapping fields of view per condition; each condition was repeated 3 times. (*N*=3, n=9). Fluorescent UTP incorporation into DMVs is inhibited by co-incubation with remdesivir triphosphate, and is resistant to RNase digestion.

Figure S7.

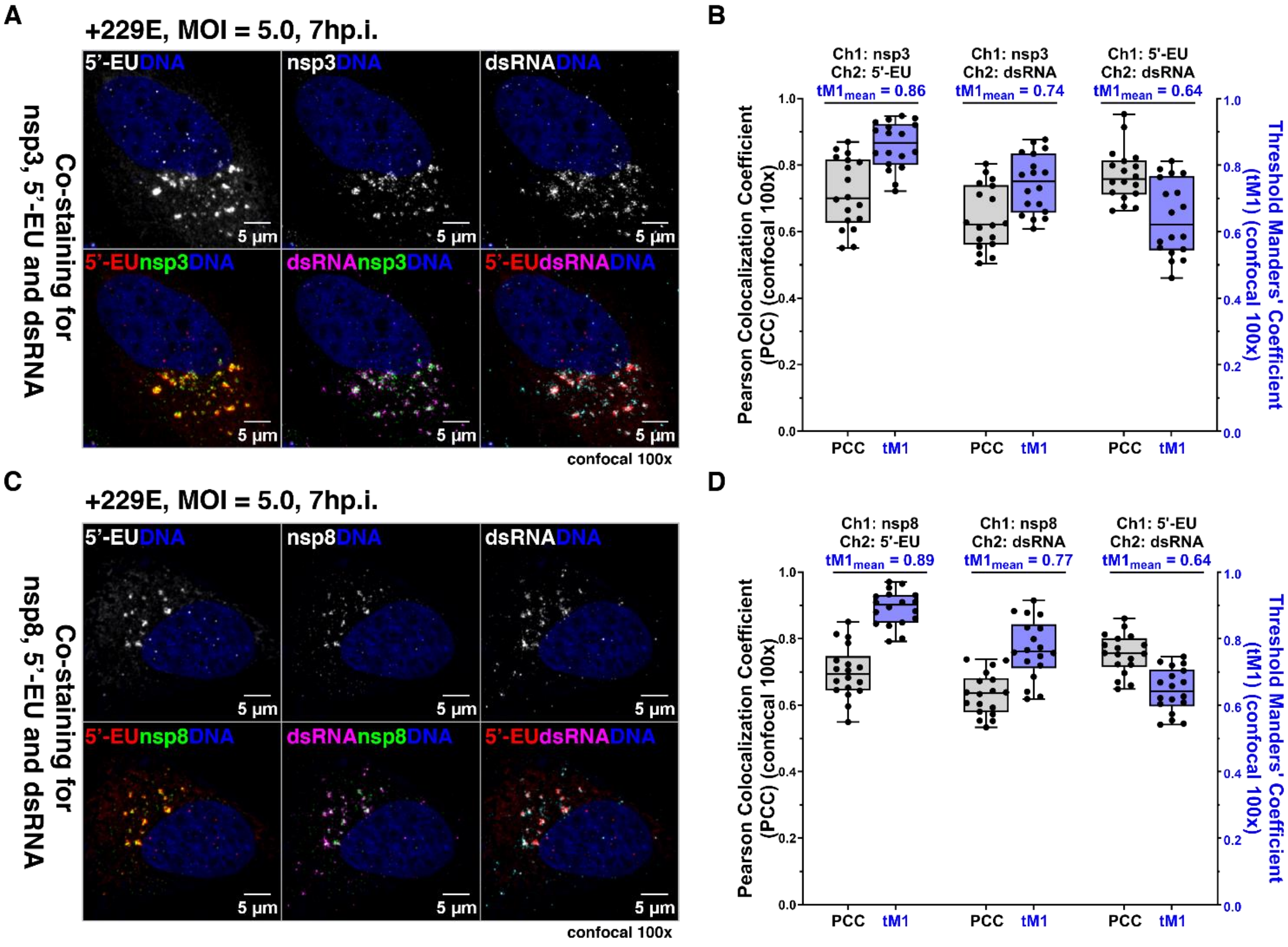

Fig. S7. Nsp3 and nsp8 overlap with DMVs at early infection stages

(A) HeLa cells infected with hCoV-229E (MOI = 5.0, 7hp.i.) were labeled with EU and were then fixed. The cells were reacted by click chemistry with biotin-azide, followed by fluorescent streptavidin, and co-staining with dsRNA probe and anti-nsp3 antibodies. The stained cells were imaged by confocal microscopy (100x). Nascent RNA labeled with EU is closely associated with nsp3 and dsRNA. **(B)** Pearson (left y-axis, gray) and Manders' coefficients (right y-axis, blue) were quantified for each pair of markers in the experiment in (A). Percentage of nsp3 (Ch1) signal that overlaps with EU or dsRNA (Ch2) was calculated using threshold Manders' coefficient (tM1). Six non-overlapping fields were analyzed for each co-detected pair, and each staining condition was repeated 3 times (*N*=3, n=18). Majority of the nsp3 signal at 7hp.i. overlaps with EU or dsRNA. **(C)** As in (A), but with staining for nsp8. **(D)** The experiment in (C) was analyzed as in (B). Majority of the nsp8 signal at 7hp.i. overlaps with EU or dsRNA.

Figure S8.

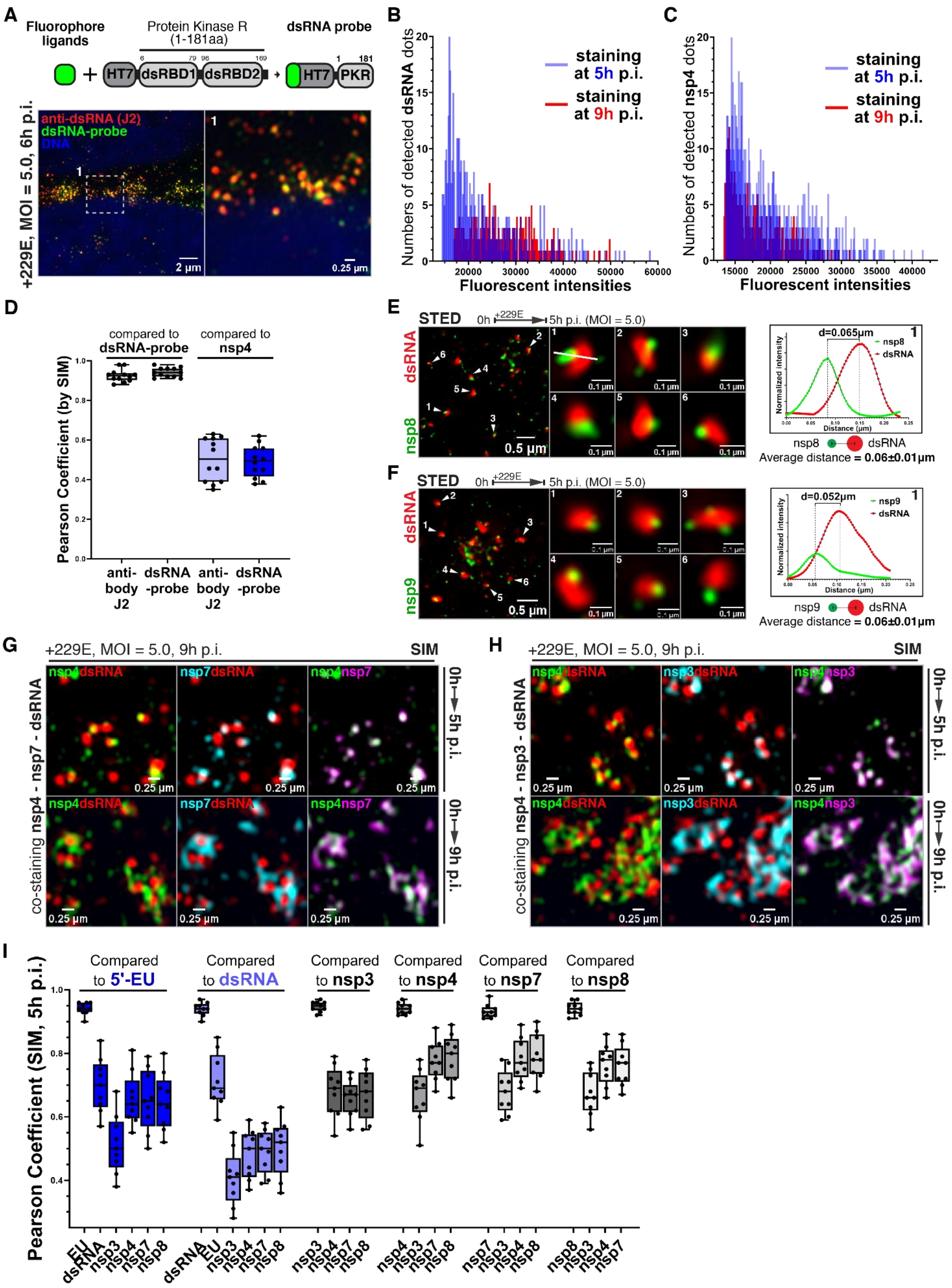

Fig. S8. High resolution imaging of dsRNA, nsp8 and nsp9

(A) Top: schematic of the dsRNA-specific probe. Bottom: hCoV-229E-infected HeLa cells (MOI = 5.0, 6hp.i.) were fixed and co-stained with the dsRNA probe and anti-dsRNA antibodies (J2). **(B and C)** As in (A), but cells co-stained with dsRNA-probe and anti-nsp4 antibodies at different post infection time points. Average intensities of dsRNA and nsp4 signals at DMVs were assigned to 300 bins, and the number of DMVs in each bin were quantified. The dsRNA intensity profile (but not nsp4) shifts from a peak at 5 hours to an even distribution at 9 hours. **(D)** As in (A-C), showing Pearson coefficients for each indicated co-detected pair. Four non-overlapping fields of view were analyzed per condition and each condition was repeated 3 times. (*N*=3, n=12). **(E, F)** As in (A), but cells fixed at 5hp.i., and co-stained with anti-nsp8 (E) or anti-nsp9 (F) antibodies and dsRNA probe, then imaged by stimulated emission depletion microscopy (STED). Middle panels show zoomed-in views of the DMVs indicated in the image on the left. The white line in middle panel 1 was used for linescan analysis (right panel), to measure distance between nsp8 and dsRNA signals. **(G and H)** As in (B and C), but co-stained with anti-nsp4, anti-nsp7, and dsRNA probe (G); or anti-nsp4, anti-nsp3 and dsRNA probe (H). **(I)** hCoV-229E-infected HeLa (MOI = 5.0, 5hp.i.) cells were fixed, permeabilized and co-stained with all possible double combinations of nsp3, nsp4, nsp7, nsp8, dsRNA and nascent RNA (EU). The cells were imaged by SIM and Pearson coefficients were quantified for all pairs. Three non-overlapping fields were analyzed for each co-detected pair, and each staining condition was repeated 3 times (*N*=3, n=9). All nsps except nsp3 strongly co-localize with EU-labeled RNA, while showing less co-localization with dsRNA. Nsp3 is an outlier, localizing away from all the other markers above.

Figure S9.

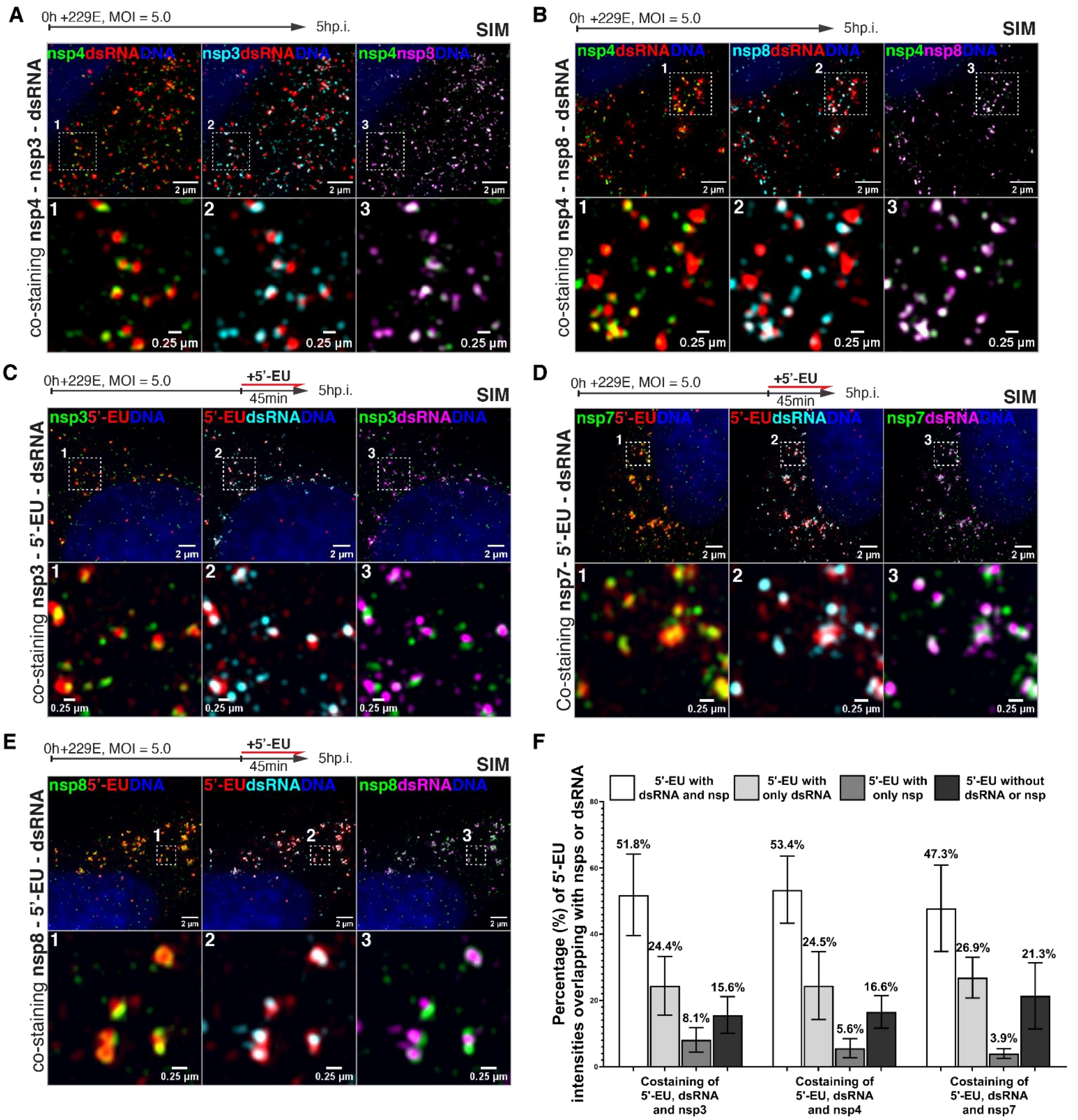

Fig. S9. Nsp7 and nsp8 are closely associated with both dsRNA and nascent viral RNA

(A) hCoV-229E-infected HeLa cells (MOI =5.0, 5hp.i.) were fixed and permeabilized, co-stained with fluorescently labeled dsRNA probe, anti-nsp3 and anti-nsp4 antibodies, and then imaged by structural illumination microscopy (SIM). **(B)** As in (A), but with staining for nsp8. **(C)** hCoV-229E-infected HeLa cells (MOI =5.0, 5hp.i.) were labeled with EU, then fixed and permeabilized. The cells were reacted by click chemistry with biotin-azide, followed by staining with fluorescent streptavidin. The cells were co-stained with dsRNA probe and anti-nsp3 antibodies, then imaged by structural illumination microscopy (SIM). **(D)** As in (C), but with staining for nsp7. **(E)** As in (C), but with staining for nsp8. **(F)** hCoV-229E-infected HeLa cells (MOI = 5.0, 5hp.i.) were fixed, permeabilized and co-stained for nascent RNA (5'-EU), dsRNA and nsps, as indicated. The cells were imaged by SIM. Percentage of 5'-EU that only overlaps with dsRNA, only overlaps with nsps, or does not overlap with either dsRNA or nsps was calculated using Manders' coefficient. Majority of nascent RNA co-localizes with both dsRNA and nsps. Three non-overlapping fields of cells were analyzed for each condition, and each condition was repeated 3 times. (*N*=3, n=9).

Figure S10.

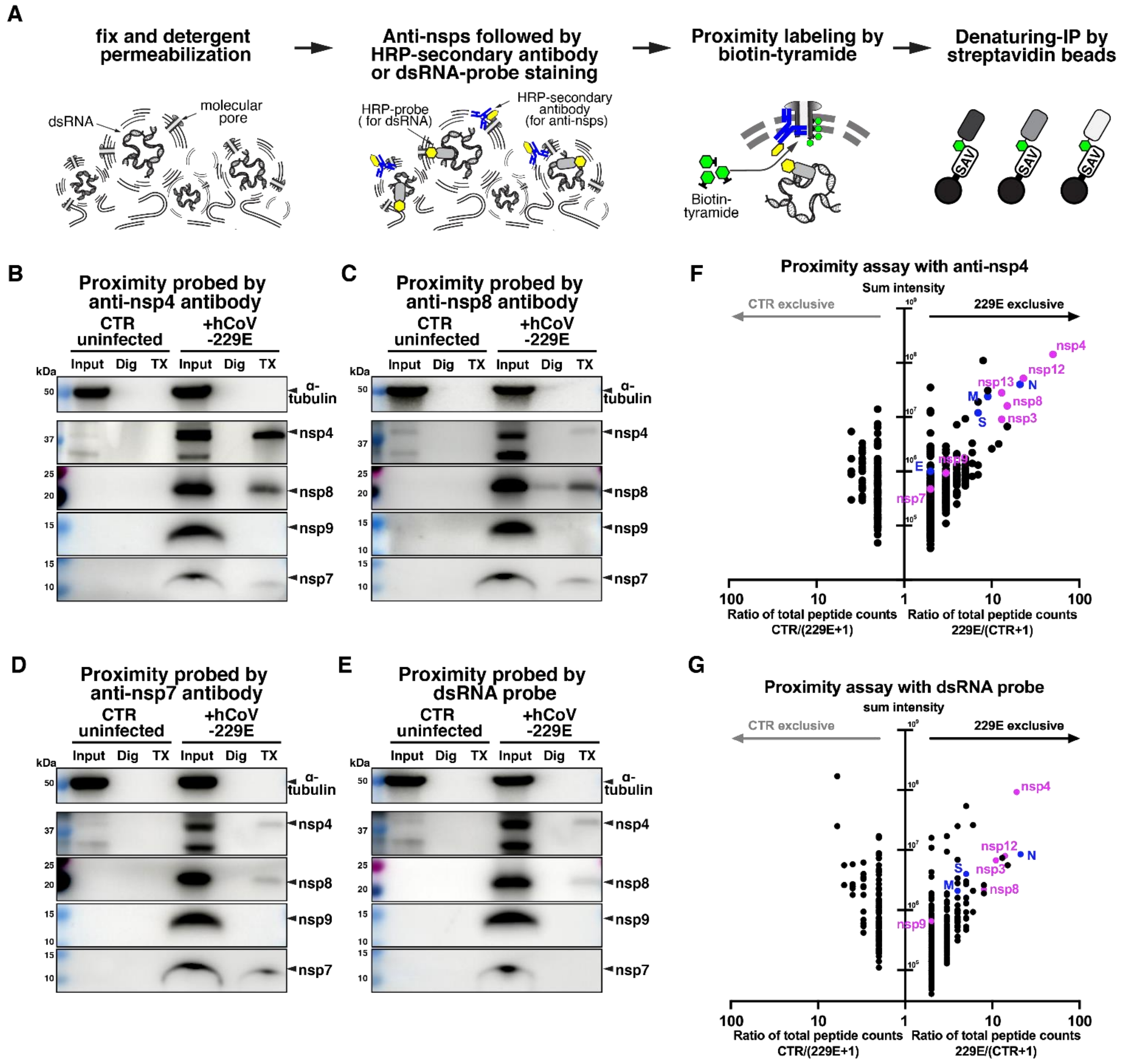

Fig. S10. Proximity detection of the replicase complex by anti-nsp4 antibodies and dsRNA probe

(A) Schematic of proximity detection in DMVs. HeLa cells infected with hCoV-229E (MOI = 5.0, 7hp.i.), are lightly fixed and permeabilized, then stained with antibodies against nsp4, nsp7 or nsp8, or dsRNA probe. Primary antibodies are detected with HRP-conjugated secondary antibodies. The dsRNA probe is directly conjugated to HRP. The cells are then reacted with biotin-tyramide, and biotinylated viral nsps are affinity purified on streptavidin beads and analyzed. **(B-E)** Cells were processed as described in (A) for nsp4 (B), nsp8 (C), nsp7 (D), or dsRNA (E). Affinity-purified biotinylated material was analyzed by SDS-PAGE and immunoblotting with antibodies against nsp4, nsp7 and nsp8. **(F-G)** Cells were processed as in (A) for nsp4 (F) or dsRNA (G). Affinity-purified biotinylated material was analyzed by LC-MS/MS. Ratios of total peptide counts in viral-infected samples were calculated by their detected numbers in viral-sample over their detected numbers in control-sample plus one [229E/(CTR+1)]. Ratio of peptide counts for each detected protein (x-axis) was plotted against peptide intensity (y-axis).

Figure S11.

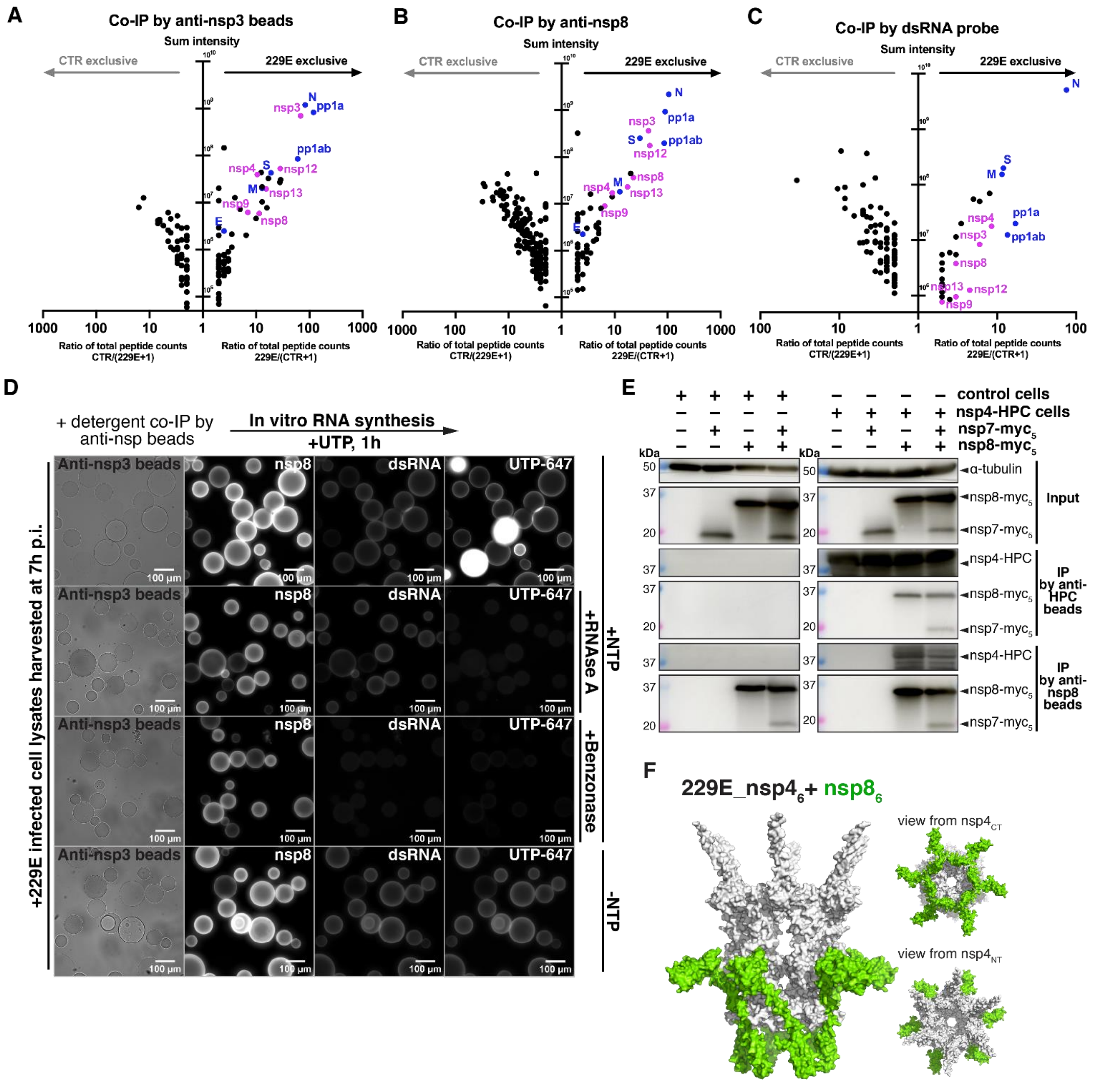

Fig. S11. A transcriptionally active replicase-pore complex

(A-C) hCoV-229E-infected HeLa cells (MOI = 5.0, 7hp.i.) were lysed, and nuclear pellets were solubilized and subjected to immunoprecipitation with beads conjugated to anti-nsp3 antibodies (A), anti-nsp8 antibodies (B), or dsRNA probe (C). Material isolated on beads was eluted and analyzed by LC-MS/MS. Ratios of total peptide counts in viral-infected samples were calculated by their detected numbers in viral-sample over their detected numbers in control-sample plus one [229E/(CTR+1)]. Ratio of peptide counts for each detected protein (x-axis) was plotted against peptide intensity (y-axis). **(D)** As in (A), but isolated beads were incubated with NTPs and fluorescent UTP, followed by incubation with the indicated enzymes. The beads were then fixed and stained for dsRNA and nsp8. **(E)** Left: HEK293 cells expressing myc-nsp7, myc-nsp8 or both, were lysed and subjected to immunoprecipitation with anti-HPC or anti-nsp8 antibodies, followed by SDS-PAGE and immunoblotting. Nsp7 and nsp8 interact. Right: as on the left, but with HEK293 cells stably expressing nsp4-HPC. Nsp4 immunoprecipitates with nsp8 and with nsp7-nsp8. **(F)** ColabFold-predicted structure of a six-fold symmetric complex between hCoV-229E nsp4 (white) and nsp8 (green). Side view (left) and end views (right) of the molecular surface.

Figure S12.

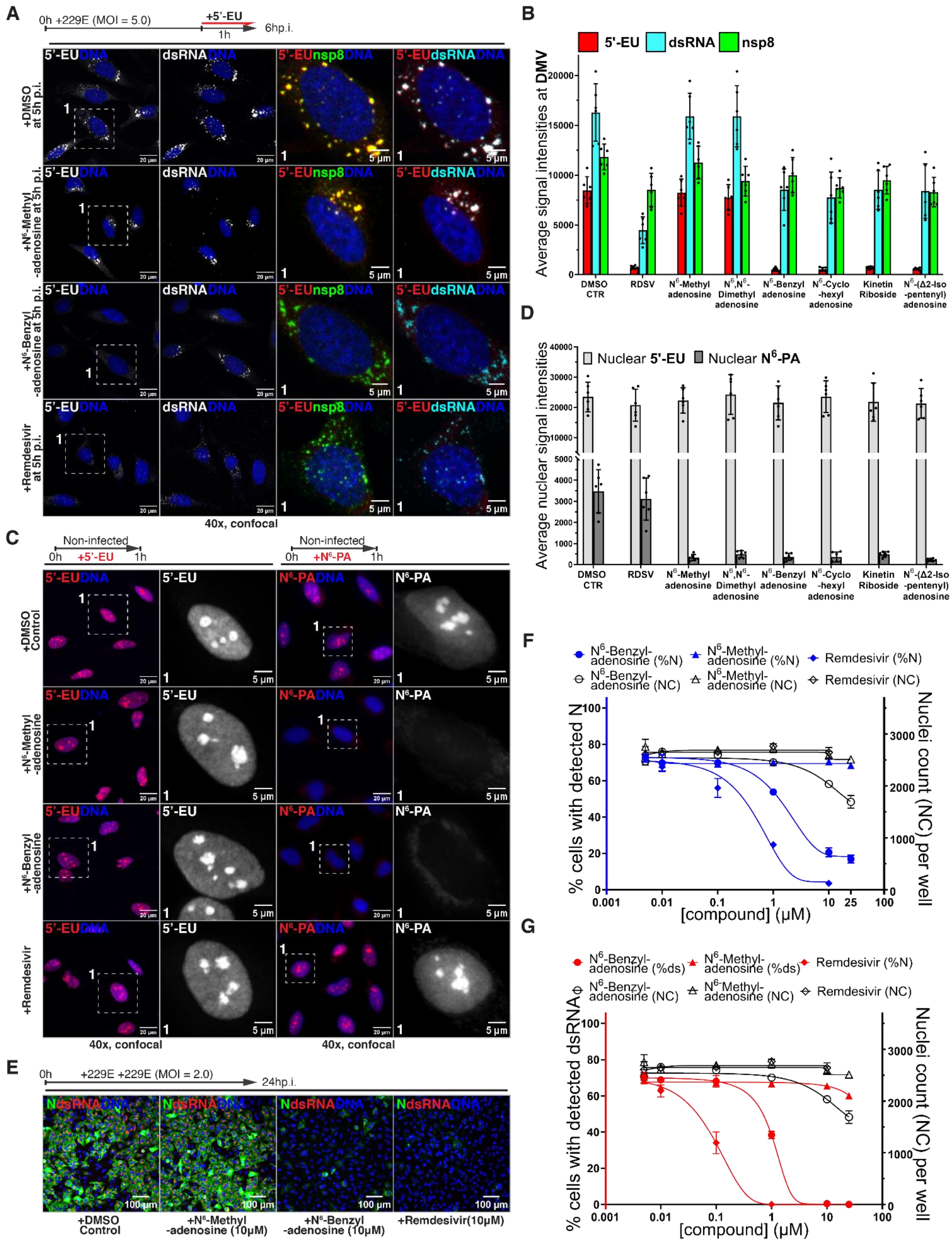

Fig. S12. Adenosine analogs with bulky N^6 modification inhibit viral RNA synthesis and replication

(A) hCoV-229E-infected HeLa cells (MOI = 5.0) were co-incubated with EU and the indicated nucleoside analogs between 5-6h p.i.; and then fixed and processed for detection of EU, dsRNA, and nsp8. **(B)** As in (A), but with additional nucleoside analogs. Average intensities of EU, dsRNA or nsp8 at DMV were quantified. Three non-overlapping fields of view were analyzed per condition, and each condition was repeated twice. (*N*=2, n=6). **(C)** Left: HeLa cells were co-incubated with EU and the indicated nucleoside analogs for 1h, fixed and then stained by click reaction with a fluorescent azide. Right: As on the left, but using N^6-propargyl-adenosine (N^6-PA) instead of EU. **(D)** As in (C), but with additional nucleoside analogs. Average intensities of nuclear EU or N^6-PA were quantified. Three non-overlapping fields of view were analyzed per condition and each condition was repeated 2 times. (*N*=2, n=6). **(E)** hCoV-229E-infected HeLa cells were incubated with the indicated compounds for 24hrs, followed by staining with anti-nucleocapsid (N) antibodies and dsRNA probe. **(F and G)** As in (E), but with a dose-response for the indicated compounds. Cells positive for N (F) or for dsRNA (G) were quantified. N^6-benzyl-adenosine inhibits viral replication while N^6-methyl-adenosine does not.

Figure S13.

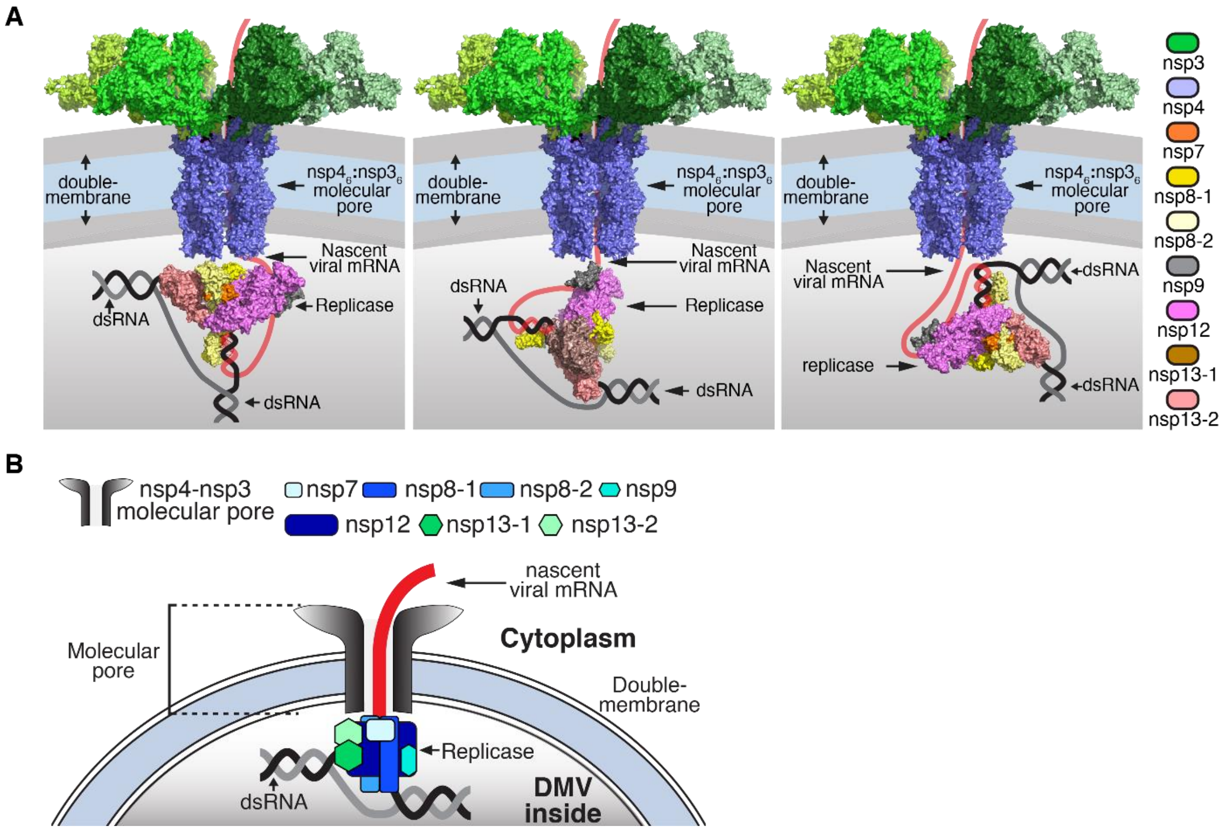

Fig. S13. Models of a transcribing replicase-pore complex inside DMV

(A) A transcribing complex formed by the replicase, pore and dsRNA. **(B)** Cartoon illustration. The replicase uses dsRNA as a template, and the nascent viral mRNA is translocated through the nsp3-nsp4 pore to the cytoplasm.

Figure S14.

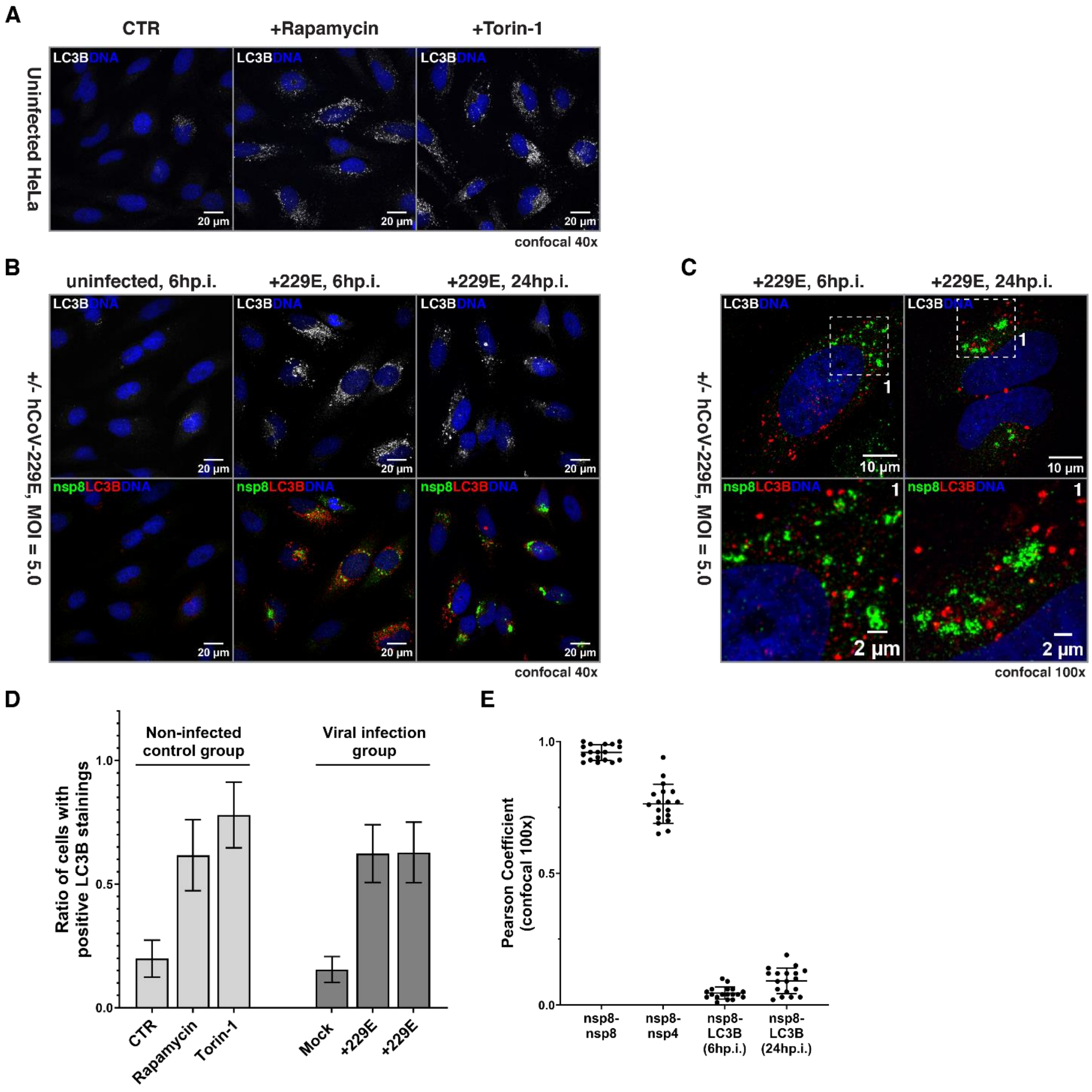

Fig. S14. The autophagy marker LC3B does not colocalize with DMVs in infected cells

(A) HeLa cells were incubated for 6hrs with rapamycin (500nM) or Torin-1 (500nM). The cells were fixed and stained with antibodies against LC3B, then imaged by confocal microscopy (40x). Autophagy induction by rapamycin and Torin-1 leads to the appearance of LC3B-positive puncta. **(B)** HeLa cells infected with hCoV-229E (MOI = 5.0) were fixed at 6 or 24hp.i. and were stained with anti-LC3B and anti-nsp8 antibodies, followed by confocal imaging as in (A). Viral infection induces LC3B puncta, but they do not overlap with the DMV marker, nsp8. **(C)** As in (B), but cells were also stained with anti-nsp4 antibodies and were imaged with a 100x objective. **(D)** Percentage of LC3B-positive cells in the experiments in (A) and (B) was quantified by image analysis. At least six non-overlapping fields were analyzed from each group and each condition was repeated 3 times (*N*=3, n=18). **(E)** Pearson coefficients were measured for the indicated pairs of markers in the experiment in (C). Six non-overlapping fields were analyzed for each co-detected pair, and each staining condition was repeated 3 times (*N*=3, n=18). Nsp8 shows strong colocalization with nsp4 but not with LC3B.

Figure S15.

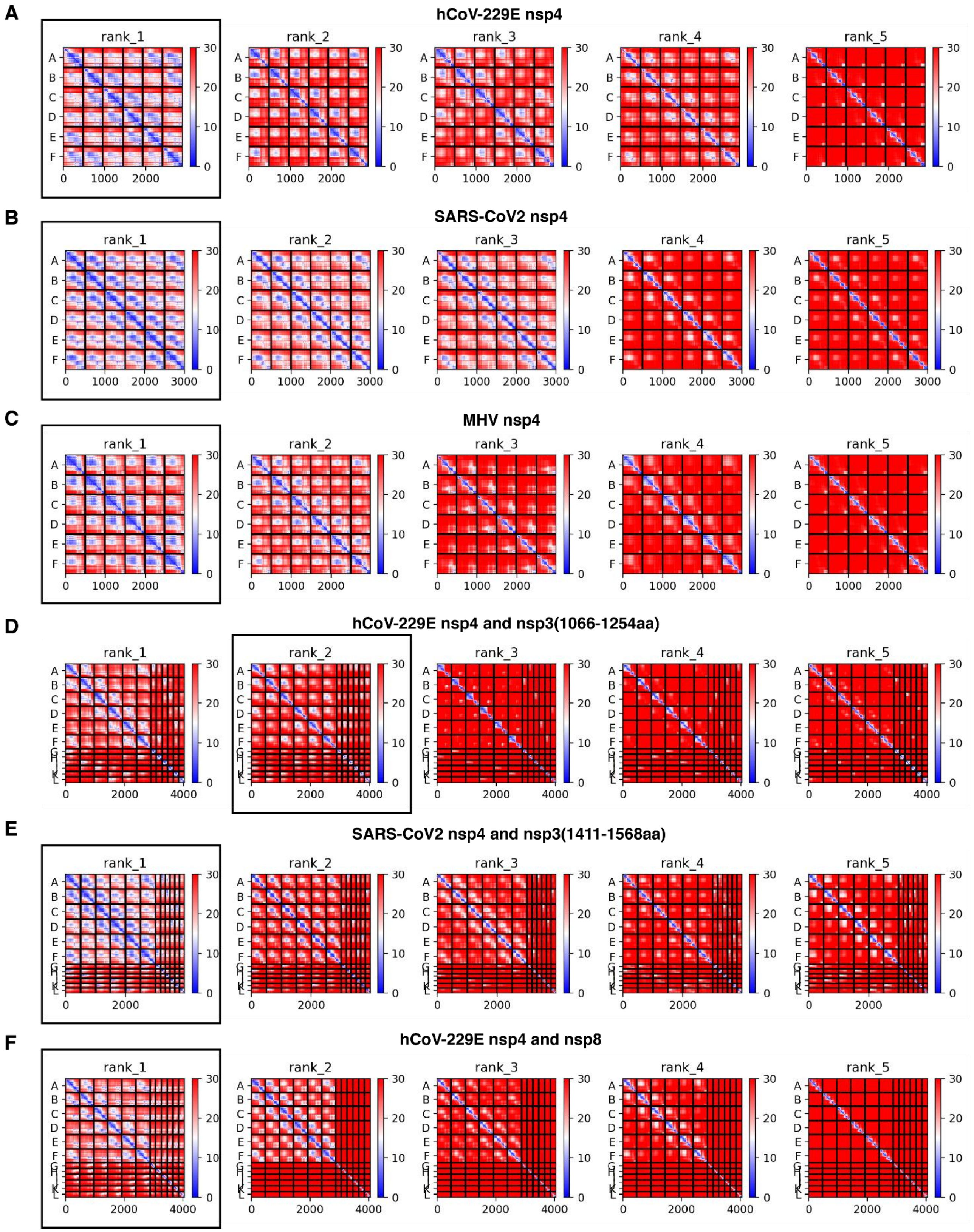

Figure S15. Predicted Alignments Errors (PAEs) for the ColabFold-predicted structures

(a) PAEs for five structures predicted by ColabFold (rank 1 to rank 5) for a hexameric hCoV-229E nsp4 complex. The six copies of nsp4 are labeled A-F. Amino acids positions are indicated on X axis. The rank 1 predicted structure was used. **(b)** As in (a), but with SARS-CoV2 nsp4. **(c)** As in (a), but with MHV nsp4. **(d)** As in (a), but with a predicted hexameric complex of hCoV-229E nsp4 + nsp3(1066-1254aa). The six nsp4 copies are labeled A-F, and the nsp3(1066-1254aa) copies are labeled G-L. The rank 2 predicted structure was used. **(e)** As in (d), but with SARS-CoV2 nsp4 + nsp3(1411-1568aa). The rank 1 predicted structure was used. **(f)** As in (d), but with hCoV-229E nsp4 + nsp8. The six copies of nsp4 are labeled A-F, and the nsp8 copies are labeled G-L. The rank 1 predicted structure was used.

Table S1. Oligo nucleotide primers used in quantitative PCR in this study

Target genes	Primer #1 (5'-3')	Primer #2 (5'-3')	Amplicon length
β-actin	CTGTGGCATCCACGAAACTA	AGTCCGCCTAGAAGCATTTG	78nt
GAPDH	GATCATCAGCAATGCCTCCT	GTCATGAGTCCTTCCACGATAC	93nt
Nucleocapsid (+ strand)	TTAGAGAGCGTGTTGAAGGTG	GTTCTGAATTCTTGCGCCTAAC	93nt
Nucleocapsid (- strand)	CAGACCCAACGACAACTACC	CTTCACACCATAGAGACCAAGAC	87nt
nsp3 (+ strand)	GCCTTACAGGGTAGATGGTAAA	CCAAAGTAAGCATAGAGTCAGT	104nt
nsp3 (- strand)	CACACAGCGACTACTGTTTGG	GTGATTCGTGTGGTGATTCAC	104nt
